# Immediate systemic neuroimmune responses following spinal mobilisation and manipulation in people with non-specific neck pain: a randomised placebo-controlled trial

**DOI:** 10.1038/s41598-023-39839-3

**Published:** 2023-08-07

**Authors:** Ivo J. Lutke Schipholt, Michel W. Coppieters, Martine Reijm, Hetty J. Bontkes, Gwendolyne G. M. Scholten-Peeters

**Affiliations:** 1 Department of Human Movement Sciences, Faculty of Behavioural and Movement Sciences, Vrije Universiteit Amsterdam, Amsterdam Movement Sciences, Program Musculoskeletal Health, Van Der Boechorststraat 9, 1081BT Amsterdam, The Netherlands; 2 Laboratory Medical Immunology, Department of Clinical Chemistry, Amsterdam University Medical Centre, Location VUmc, Amsterdam, The Netherlands; 3 School of Health Sciences and Social Work, Menzies Health Institute Queensland, Griffith University, Brisbane & Gold Coast, Australia

**Keywords:** Cytokines, Diseases, Musculoskeletal system, Therapeutics, Pain management

## Abstract

Spinal mobilisation/manipulation is a common intervention for spinal pain, yet the working mechanisms are largely unknown. A randomised placebo-controlled trial was conducted to (1) compare the immediate neuroimmune responses following spinal mobilisation/manipulation and placebo spinal mobilisation/manipulation; (2) compare the immediate neuroimmune responses of those with a good outcome with those of a poor outcome following spinal mobilisation/manipulation; and (3) explore the association between neuroimmune responses and pain reduction. One hundred patients were randomly allocated to spinal mobilisation/manipulation or a placebo mobilisation/manipulation. Primary outcomes were whole blood in-vitro evoked released concentrations of IL-1β and TNF-α measured 10 min and 2 h after the intervention. Immediate effects were studied because successful mobilisation/manipulation is often associated with immediate pain reduction, and immediate neuroimmune responses are less affected by potential confounders than long-term responses. Secondary outcomes included multiple systemic inflammatory marker concentrations, phenotypic analysis of white blood cells and clinical outcomes. Outcomes were compared between the experimental and placebo group, and between people with a good and poor outcome in the experimental group. Estimates of intervention effects were based on intention-to-treat analyses, by using linear mixed-effect models. Although there was a substantial difference in pain reduction between groups (mean (SD) difference visual analogue scale: 30 (21) mm at 10 min and 32 (21) mm at 2 h (p < 0.001) in favour of mobilisation/manipulation, there were no differences in primary outcomes between groups or between people with a good and poor outcome (p ≥ 0.10). In conclusion, possible neuroimmune responses following spinal mobilisations/manipulation cannot be identified at a systemic level. Future research may focus on longer treatment duration and more localised neuroimmune responses.

## Introduction

Spinal mobilisations/manipulation is commonly used in the management of people with spinal pain^1^. Systematic reviews have shown that spinal mobilisation/manipulation improve pain and function at short term follow-up^2,3^. It may positively affect several neurophysiological, biomechanical and psychological responses^4,5^. However, the mechanisms mediating the effects of spinal mobilisation/manipulation on pain reduction remain poorly understood^5,6^.

Systemic inflammatory markers, such as high sensitive c-reactive protein (hsCRP) and tumor necrosis factor (TNF)-α may be elevated in people with persistent neck pain^7^ and radicular pain^8^. These immune responses may be associated with pain intensity and perceived recovery, suggesting that spinal pain encompasses inflammatory components^7,9^. As systemic inflammation is upregulated and associated with clinical outcomes in people with non-specific neck pain, further interventional research is warranted to provide insights whether and how systemic inflammation can be reduced^9^. It has been proposed that spinal mobilisation/manipulation stimulates the hypothalamic–pituitary–adrenal-axis and the sympathetic-adrenal-medullary-axis, and subsequently promotes autonomic nervous system and immune system interactions diminishing systemic inflammation^10,11^.

Several studies have explored the effects of joint mobilisation/manipulation on neuroimmune responses in people with low back pain^12–15^. Several non-randomised trials revealed that spinal mobilisation/manipulation attenuated inflammatory immune responses^12,13,15^. These studies showed an association in time between in-vitro evoked-released reduced levels of CC motif chemokine ligand (CCL)-3, CCL-4, TNF-α, IL-6, soluble TNF receptor type (sTNFR)-2, ex-vivo serum hsCRP, and pain intensity in patients who received spinal mobilisations/manipulation^12,13,15^. However, the results of these studies have to be interpreted with caution as they used modest sample sizes^12,13,15–17^, narrow selections of inflammatory markers^13^, lacked correction for potential confounding variables^12,13,15–17^, did not investigate the associations between inflammatory markers and clinical outcome^12,13,15–17^, or failed to use a placebo and randomised control group^12,13,15–17^.

To our knowledge, only one randomised controlled trial explored the association between systemic neuroimmune responses, spinal mobilisation/manipulation and pain reduction in patients with non-specific neck pain^17^. In that study, several sessions of thoracic manipulations reduced the ex-vivo serum level of interleukin (IL)-1β compared to a placebo intervention^17^. Considering the methodological shortcomings in the current literature and that only one study was conducted in people with non-specific neck pain, properly conducted placebo-controlled randomised trials addressing these weaknesses are warranted.

Confounding factors, such as variations in medication use, medical co-morbidities, alcohol usage, psychological stress, sleep, and cyclical variations in neuro-endocrine-immune responses, complicate the study of long-term effects of interventions on systemic inflammation. Omitting these confounders may induce significant bias as these factors may induce variability over time^18,19^. However, accounting for these multiple factors requires very large sample sizes^19,20^. Studying immediate treatment effects which are affected by fewer confounders is therefore a valuable first step. Therefore, this study aimed to (1) compare the immediate neuroimmune responses following spinal mobilisation/manipulation and placebo spinal mobilisation/manipulation; (2) compare the immediate neuroimmune responses of those with a good outcome (i.e., substantial pain relief) with those with a poor outcome (i.e., minimal or no pain relief) following spinal mobilisation/manipulation; and (3) assess the association between immediate neuroimmune responses and pain reduction following spinal mobilisation/manipulation.

## Methods

### Design

This study was a placebo-controlled randomised trial with immediate follow-up. The study protocol has been preregistered (https://trialsearch.who.int with study ID: NTR6961) and published^21^. The Medical Ethics Committee of Amsterdam University Medical Centre, location VUmc, approved the study (Approval number: 2018.181) and it was registered at trialregister.nl with study ID: NL6575. All participants provided written informed consent prior to participating in the trial and which was performed in accordance with the declaration of Helsinki. Data were collected between February 2019 and January 2022. The study is reported according to the CONSORT^22^ statement and TIDieR checklist^23^.

### Participants

People with non-specific neck pain were recruited from General Practitioner clinics and primary care physiotherapy practices in The Netherlands. The first author (ILS) enrolled the participants. People aged between 18 and 65 years, with a minimum pain intensity of 40/100 on a Visual Analogue Scale (VAS), and at least six weeks of non-specific neck pain were eligible to participate in the trial^24^. Exclusion criteria were: treatment for the current neck pain episode during the preceding two weeks, taken non-steroidal anti-inflammatory medication within the past 7 days, having a known comorbid condition with immune/endocrine dysfunction (e.g., ankylosing spondylitis), medical red flags suggestive of serious pathology^1,25^, or a diagnosed psychological condition (e.g., depression). A more detailed description of the selection criteria is presented in our protocol paper^21^.

For longitudinal analyses with three time points (baseline and 2 follow-up times), 80% power to detect a mean (SD) difference of 550 (933) pg/ml for TNF-α, with a two-sided significance level, a correlation of 0.6 among repeated measures and a ratio between the experimental group and control group of 0.33, 91 participants were required. Allowing a ~ 10% drop-out rate, 100 participants were recruited^16,26^.

### Randomisation and blinding

People with non-specific neck pain (n = 100) were randomly allocated to the experimental group (n = 75) or the placebo control group (n = 25). A computer random number generator was used to select blocks with block sizes of 4 and 8, with an allocation ratio 3:1. An independent person generated the random allocation sequence and allocated in a concealed manner the participant at the start of the treatment. Based on the immediate changes in pain intensity (i.e., at 10 min and 2 h following the intervention), measured using the visual analogue scale (VAS 0–100; pain at rest), participants in the experimental group were categorised into those with a good outcome (i.e., ≥ 50% improvement in pain intensity at both time points), a poor outcome (i.e., ≤ 20% improvement in pain intensity at both time points) or an unclear outcome (not fitting the criteria for a good or poor outcome)^27^. The participants, outcome assessors and laboratory personal were unaware of treatment allocation and outcome categorisation during data collection. The clinicians were also blinded for good/poor outcome categorisation. The researchers who performed the statistical analyses and interpretation were blinded for treatment allocation and good/poor outcome categorisation. After consensus was reached on the interpretation of the results, treatment allocation and good/poor outcome categorisation were revealed.

### Interventions

**Figure 1:**
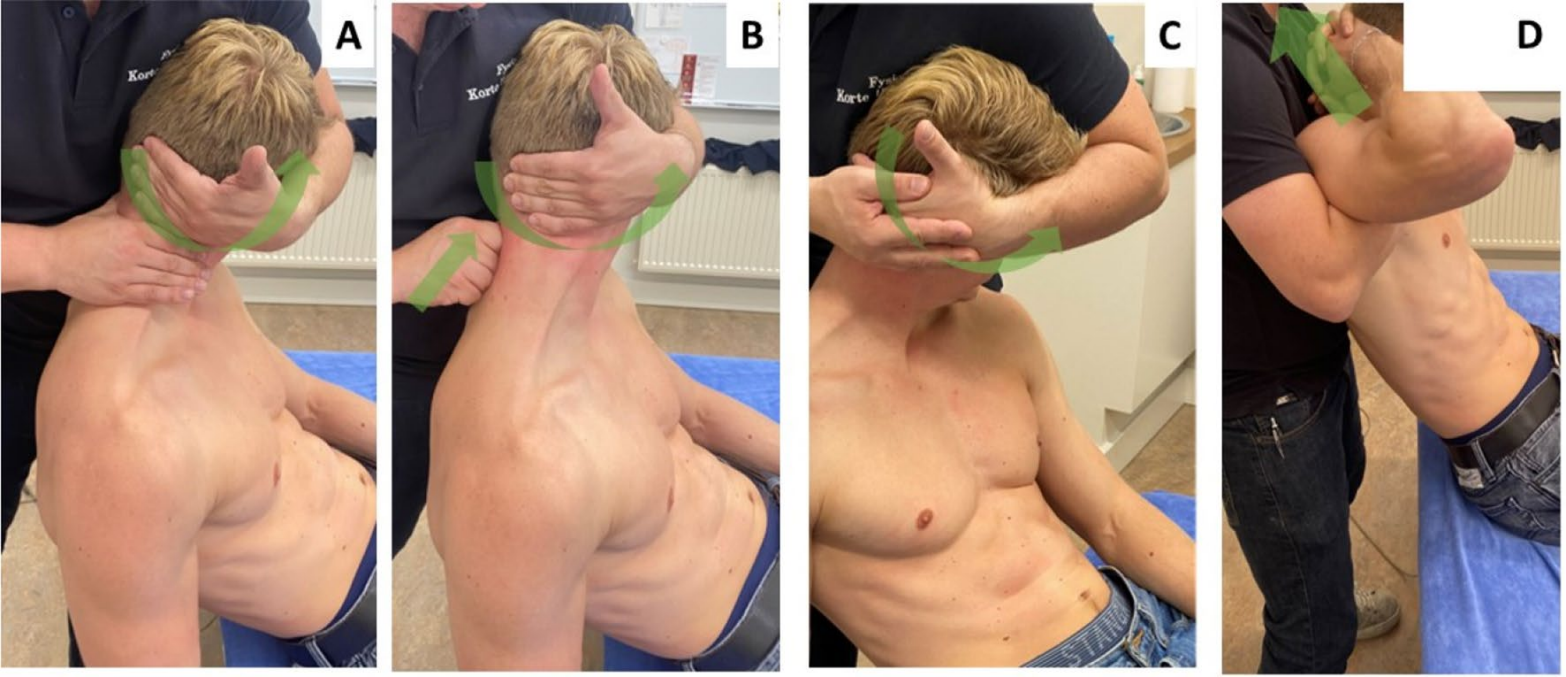
Spinal mobilisation and manipulation techniques. Depending on the identified painful segmental levels, the clinician selected from different cervical mobilisation techniques (**A**–**C**); (**A**) Mobilisation targeting the atlanto-axial joint*s*. The cervical segments below the second cervical vertebrae was submaximal rotated and lateroflexed. With the clinician’s hypothenar region of the hand over the structures overlying the arcus of the first vertebrae, the clinician translated the head *further in rotation*^49^. (**B**) Segmental zygapophyseal joint mobilisation (C2 to C7; the image shows the technique for C3-C4). First, the occipital-atlanto-axial joint was maximally rotated in the direction of the joint being mobilised. Subsequently, the head is moved to extension, lateroflexion and rotation until pressure from the thumb was felt. This technique was repeated on the lower level until the painful cervical segment was reached (C3-C4). Next, on the painful cervical segment, in *cranio-ventral direction*, pressure was given^49^. (**C**) Mobilisation technique targeting the occipital-atlanto-axial joint*s*. The clinician’s hypothenar region was placed against the mastoid process. C2 to C7 were submaximally locked in flexion, rotation and lateroflexion. The head was then moved in a *medio-caudal direction*^49^. (**D**) Spinal manipulation technique targeting the cervico-thoracic junction. The participant was seated on a treatment table. The height of the table was adjusted to the level of the clinician’s abdomen. The participant’s hands was placed on the back of their head (one hand placed over the other, rather than with interlocking fingers), and with the shoulders slightly retracted. The clinician’s hands was placed over the hands of the participant, with the clinician’s forearms ventral the shoulder of the participant. Then, a high-velocity, low-amplitude movement was applied in a *dorsal-cranial direction*^49^. Green arrows represent the mobilisation (**A**–**C**) or manipulation (**D**) direction.

The experimental group received spinal mobilisations at the painful and restricted cervical level(s) and a spinal manipulation at the cervico-thoracic junction. Spinal mobilisation consisted of low-velocity, low-amplitude segmental cervical mobilisations (Fig. 1A–C), three series of oscillations (~ 1 Hz) for 30 s; with 30 s rest in between the series. The spinal manipulation was a high-velocity, low-amplitude distraction manipulation at the cervico-thoracic junction (Fig. 1D)^28^. The aim was to decrease pain intensity and improve cervical range of motion. The control group underwent a placebo mobilisation/manipulation. Procedures, including the instructions for the control group were identical as for the experimental group, except that the clinician only applied hand contact and no pressure or movement occurred. All interventions were delivered in a primary care physiotherapy practice by two physiotherapists with > 10 years relevant experience in spinal mobilisation/manipulation. The standardised instruction and treatment techniques can be found in Fig. 1 and are described in detail elsewhere^21^.

### Outcome measures

#### Primary outcomes

The immediate differences in whole blood in-vitro evoked release levels of IL-1β and TNF-α after between the experimental and control group were the primary outcomes. Fasting heparinised samples of peripheral blood, taken between 08:00 and 09:00 A.M. and processed after 4 h, were used for whole blood culture. To induce the production of these cytokines, whole blood cultures were cultivated for 24 h at 37 °C in a humified 5% CO_2_ incubator with the presence of lipopolysacharide from Escherichia coli serotype 055:B5 (LPS; Sigma) at concentrations of 1 ng/ml (low dose stimulation) and 10 µg/ml (high dose stimulation). Following the incubation period, supernatants were centrifuged, aliquoted and frozen at − 80 °C until the analyses were performed. The levels of in-vitro IL-1β and TNF-α were determined using a custom-made U-plex (MSD, Maryland, United States) conforming to manufacturer recommendations. Supernatants were diluted 100-fold prior to testing.

#### Secondary outcomes

**Figure 2:**
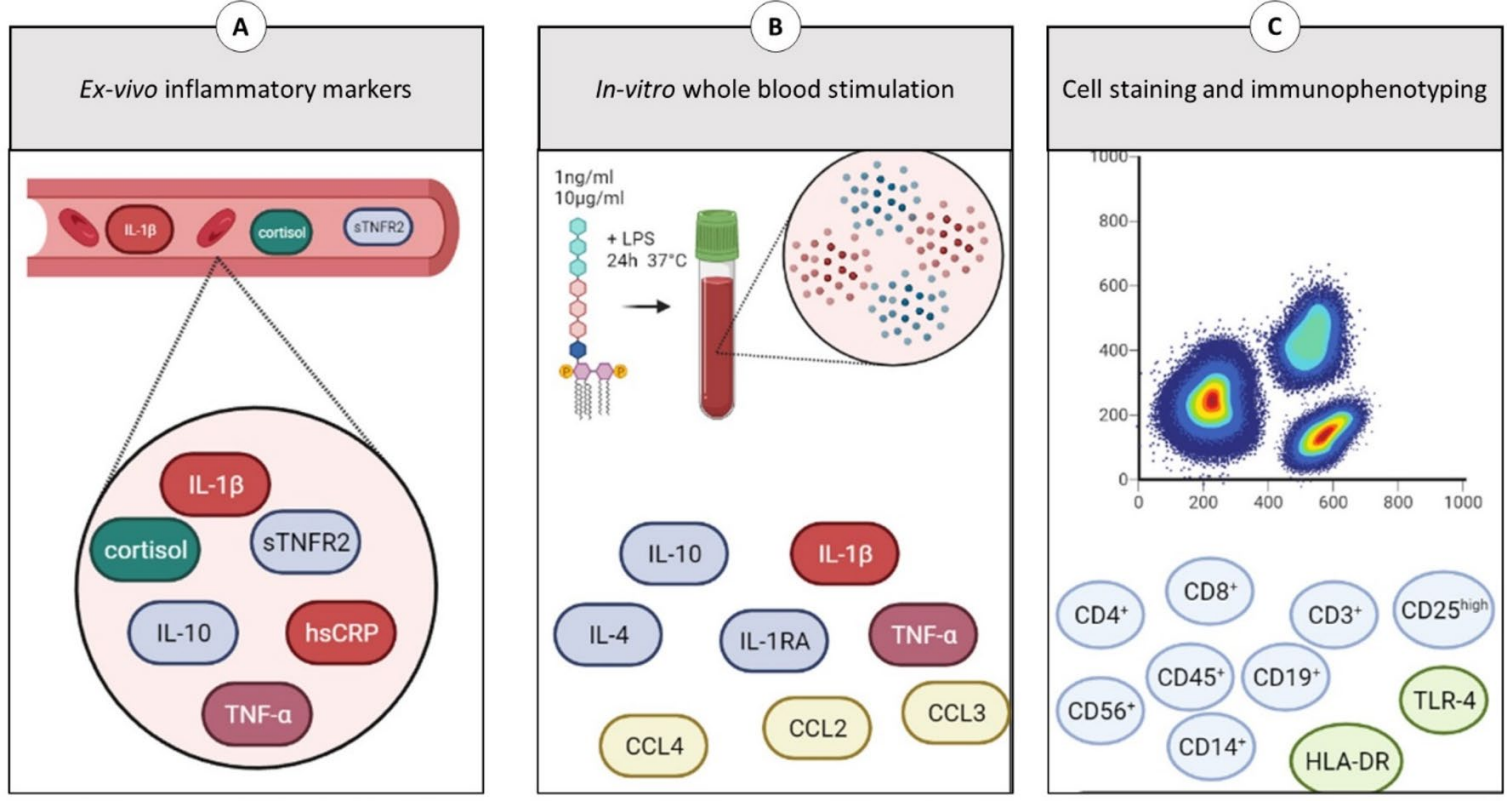
Neuroimmune parameters. (**A**) Measured using multianalyte assay Ella (R&D systems, Minneapolis, United States) Cardiac C-Reactive Protein (Latex) High Sensitive using R oche/Hitachi cobas c systems. Markers TNF-α (Inter-assay coefficient of variation: 4.27%), sTNF-R2 (5.78%), IL-1β (4.97), IL-1RA (7.20%) directly from blood samples measured using multianalyte assay Ella (R&D systems, Minneapolis, United States). Aliquots of blood samples to determine ex-vivo levels of inflammatory markers were stored at − 80 °C after centrifugation for 10 min at 1530*g*. (**B**) Stimulated for 24 h at 37 °C, in a humidified 5% CO_2_ incubator, with lipopolysaccharide (LPS) from *Escherichia coli O55:B5* at a concentration of 1 ng/ml and 10 µg/ml. Determined using a custom-made U-plex (MSD, Maryland, United States) Whole blood was stimulated with high dose (10 µg/ml (HD-LPS)) or low dose (1 ng/ml) LPS. Supernatant was diluted 100-fold and tested for TNF-α (Inter-assay coefficient of variation: TNF-α (7%), IL-1β (12.7%), IL-1RA (10.6%), IL-10 (22%), CCL2, (8.4%), CCL3 (12.7%), CCL4 (12.9%) using the above-mentioned U-plex. (**C**) Determined by 10-color flowcytometry (FCM): CD45+ = General Leukocyte marker; CD3+ = T-cell marker; CD3+CD4+ = CD4+ T-helper marker; CD3+CD4+CD25hi = T-regulator cell marker; CD3+CD8+ = Cytotoxic T-cell marker; CD3-CD56+ = Natural Killer cell marker; CD19+ = B-cell marker; CD14+ = monocyte marker; HLA-DR = activation marker for T-cells and monocytes; TLR-4 = Toll-like receptor 4 marker. CD25+ = activation marker for T-cells, Fluorescence-activated cell sorting (FACS) staining was used for cell surface staining of mononuclear cells using a whole blood staining protocol and red blood cell lysis using optilyse B conform manufacturer recommendation (Beckman Coulter, Brea, CA). For quantification of lymphocyte subsets Trucount tubes were used (BD Biosciences, Franklin Lakes, NJ). The following monoclonal antibodies were used: CD8-APC-AF700 (B9.11), CD19-ECD (J3-119), CD56-PC7 (N901) all from Beckman Coulter; HLA-DR-FITC (G46-6), CD14-APC (M5E2), TLR4-PE (TF901) all from BD Pharmingen (San Diego, CA) and CD3-APC (SK7), CD4-APC-H7 (SK3), CD25-PE (2A3), CD45-PerCP (2D1) all from BD Biosciences. HLA-DR was used as activation marker for T-cells and monocytes, CD25 was used as activation marker for T-cells; TLR-4 expression was assessed on monocytes. Isotypes were used as control for these activation markers. Samples were run on FACS Gallios (Beckman Coulter) and analyzed using Kaluza (Beckman Coulter). The total number of leucocyte was determined using Z2 analyzer (Beckman Coulter). TNF-α: Tumor Necrosis Factor-α; TNF-RII: Tumor Necrosis Factor Receptor Antagonist 2; IL-1β: Interleukin-1β; IL-1RA: Interleukin-1 receptor antagonist; hsCRP: High sensitive C-Reactive Protein; IL-4: Interleukin-4; IL-10: Interleukin-10; CCL2: c–c-motif chemokine ligand 2; CCL3: c–c-motif chemokine ligand 3; CCL4: c–c-motif chemokine ligand 4; CD: cluster of differentiation.

A broad range of systemic neuroimmune responses were quantified as secondary outcomes: (a) inflammatory marker concentration following in-vitro stimulation of whole blood cells (IL-10, IL-4, IL-1RA, CCL2, CCL3 and CCL4), (b) ex-vivo serum inflammatory markers (TNF-α, IL-1β, sTNFR-R2 and IL-1RA) (c) ex-vivo serum cortisol, and (d) phenotypic analysis of blood mononuclear cells (see Appendix 1 for the gating strategies). A broad description of determination of the neuroimmune responses is presented in Fig. 2. To examine a general change in inflammatory marker production, in-vitro and ex-vivo overall inflammatory, proinflammatory, anti-inflammatory and ratio proinflammatory/anti-inflammatory indices were calculated^21^.

In addition, several psychological and behavioural self-reported questionnaires and physical tests were evaluated (Table 1). Serious adverse events related to the experimental and/or control intervention were documented.

**Table 1:** Patient characteristics variables and outcome measures.

	Baseline	10 min	2 h	2 days
Questionnaires				
Pain intensity (VAS)	✓	✓	✓	
Mental health (MH15)	✓			
General health		✓		
Perceived recovery (GPE)			✓	✓
Disability (NDI)		✓		
Sleep quality (PSQI)		✓		
Physical activity (IPAQ)		✓		
Kinesiophobia (TSK)		✓		
Central sensitization (CSI)		✓		
Depressive, anxiety, stress symptoms (DASS21)		✓		
Neuropathic pain (PAINdetect)		✓		
Placebo questions		✓		
Physical tests				
Cervical range of motion (CROM)	✓	✓	✓	
Pressure pain threshold (PPT)	✓	✓	✓	
Temporal summation	✓	✓	✓	
CROM-VAS		✓	✓	
Visceral adipose tissue (VAT)		✓		
Neuroimmune markers				
Ex-vivo levels of inflammatory markers	✓	✓	✓	
In-vitro levels of inflammatory markers	✓	✓	✓	
Blood phenotyping	✓		✓	
Ex-vivo cortisol	✓	✓		
Adverse events				
Questionnaire		✓	✓	✓

The questionnaires and ultrasound measures that were not used to assess treatment effects and that were considered to be influenced negligibly by the intervention were completed while the patient was waiting for his outcome assessment at 2-h after the intervention.

VAS: Visual Analogue Scale; MHI5: Mental Health Inventory-5; NDI: Neck Disability Index; PSQI: Pittsburg Sleep Quality Index; IPAQ: International Physical Activity Questionnaire: TSK: Tampa scale for kinesiophobia; CSI: Central Sensitisation Inventory; DASS21: Depression, Anxiety, Stress Scale 21; GPE: Global Perceived Effect scale; Placebo questions: The extent to which they agree (using a four-point Likert scale) with four statements^48^. CROM: Cervical Range of Motion; PPT: Pressure Pain Threshold, PPTs were assessed bilaterally over the mid-point trapezius (pars descendens), second metacarpal and tibialis anterior muscle.; Temporal summation: Using a pinprick 256 mN wind-up ratio were calculated bilaterally over the midpoint trapezius (pars descendens) and tibiales anterior muscle. CROM-VAS: This test consists of two parts. In part 1, the participant was asked to perform maximal active right and left cervical rotation and the degrees of rotation were reordered using the CROM device. In this position, the pain intensity was measured with the VAS following intervention. After the intervention, part 2 of the test was performed. The participant was again asked to actively rotate (left and right) to the same position as in part 1 and the pain intensity was recorded. The difference on VAS scores was the outcome of the CROM-VAS test. VAT: Visceral Adipose Tissue. Linear distance between abdominal peritoneum and ventral aspect of vertebrae will be assessed using ultrasonography. Ex-vivo levels of inflammatory markers: TNF-α, sTNFR2, IL-1β and IL-1RA. In-vitro levels of inflammatory markers: TNF-α, IL-1β, IL-1RA, IL-10, IL-4, CCL2, CCL3, CCL4. Blood phenotyping for: CD45+ = General Leukocyte marker; CD3+ = T-cell marker; CD3+CD4+ = CD4+ T-helper marker; CD3+CD4+CD25hi = T-regulator cell marker; CD3+CD8+ = Cytotoxic T-cell marker; CD3-CD56+ = Natural Killer cell marker; CD19+ = B-cell marker; CD14+ = monocyte marker; HLA-DR = activation marker for T-cells and monocytes; TLR-4 = Toll-like receptor 4 marker. CD25+ = activation marker for T-cells. Ex-vivo serum cortisol. Bold represents a significant difference between groups (p < 0.05).

### Data analysis

Normality of continuous variables was visually inspected by Q–Q plots, box plots and histograms and checked with the Kolmogorov–Smirnov test. Baseline demographic variables and clinical symptoms were compared between the groups (experimental vs. control; good vs. poor outcome in the experimental group) using independent sample t-tests or Mann–Whitney U tests for continuous variables and chi-square tests for dichotomous variables.

Changes over time in pain intensity (VAS) and physical tests, and differences between groups were assessed using linear mixed models with fixed factor (time) and covariate (group) and interaction (time*group) with an intention-to-treat approach. Data for the inflammatory markers were Ln-transformed as these were not normally distributed. The inflammatory indices were calculated on the Ln-transformed and z-score levels (based on the control group or poor outcome group) of the inflammatory markers^21^. To compare the immediate neuroimmune responses between the experimental and control group, and between the good and poor outcome group within the experimental group, linear mixed model analyses with fixed factor (time), covariate (group) and interaction (time*group) were used to detect differences between the groups at the three time points with an intention-to-treat approach. A random intercept was chosen to account for the correlated nature of multiple measurements from the same individual. The regression coefficient (B), p-value, and standard error were computed for the crude Model 1, as well as for the adjusted models. Several potential confounding factors were taken into account and statistical models were built based on technical variations (such as platenumber) and other factors that differed between the groups (Tables 1 and 2). All potential confounders were a-priori described in our protocol paper^21^. To avoid overfitting of the statistical models when performing the neuroimmune analysis, different adjusted models were computed for the in-vitro and ex-vivo analysis. For the analysis of the in-vitro neuroimmune responses three additional adjusted models were computed (model 2a, 3a and 4) and for the in-vivo neuroimmune response two additional adjusted models were computed (model 2b and 3b): *Model 2*: Model 1, normalised/1000 monocytes; *Model 3*: Model 2, adjusted for age, gender and body mass index; *Model 4*: Model 2, adjusted for plate number, function (neck disability index (NDI) score), catastrophizing (pain catastrophising scale (PCS) score), kinesiophobia (tampa scale of kinesiophobia (TSK) score) and central sensitisation (central sensitisation index (CSI) score). The additional serum ex-vivo models were: *Model 2b*: Model 1 additional adjusted for age, gender and body mass index; *Model 3b*: Model 1 additional adjusted for plate number, NDI score, PCS total score, TSK score and CSI score.

**Table 2:** Demographic and clinical characteristics.

Characteristic	Experimental	Control	Sig.	Good outcome	Poor outcome	Sig.
Age, in years	45 (13)	43 (15)	0.61^†^	45 (12)	39 (14)	0.09^†^
Gender female: N (%)	N = 48 (65%)	N = 19 (74%)	0.42^‡^	N = 20 (56%)	N = 13 (77%)	0.14^‡^
BMI, kg/m^2^	25 (4)	27 (5)	0.12^†^	26 (5)	24 (5)	0.29^†^
Pain (VAS)	51 (40–67)	59 (43–75)	0.89^†^	43 (40—60)	46 (40–68)	0.63^§^
Duration symptoms (months)	25 (6–120)	36 (5–120)	0.95^§^	60 (12–156)	19 (6–93)	0.10^§^
PAINdetect ≥ 19, %	49	44	0.83^‡^	57	41	0.27^‡^
Disability	**28 (12)**	**38 (14)**	**0.001**^**†**^	26 (12)	32 (11)	0.14^†^
Neuropathic pain %	62	48	0.29^‡^	19	12	0.75^‡^
Depressive symptom	4 (0–8)	4 (0–20)	0.44^§^	4 (0–8)	6 (2–18)	0.06^§^
Anxiety symptom	4 (1–10)	6 (2–18)	0.23^§^	4 (1–10)	5 (2–9)	0.54^§^
Stress symptom	9 (4–15)	10 (3–18)	0.95^§^	8 (4–14)	12 (3–25)	0.18^§^
Kinesiophobia (Tampa > 37), %	**19**	**39**	**0.05**^**‡**^	20	29	0.45^‡^
Pain catastrophizing (total score)	**16 (10)**	**24 (13)**	**0.001**^**†**^	16 (10)	17 (10)	0.70^†^
Pain rumination	**6 (3–8)**	**9 (5–12)**	**0.01**^**§**^	7 (3–9)	6 (4–7)	0.73^§^
Pain magnification	**2 (1–4)**	**4 (2–7)**	**0.007**^**§**^	2 (1–3)	2 (1–4)	0.90^§^
Pain helplessness	**6 (3–10)**	**11 (6–16)**	**0.008**^**§**^	6 (4–10)	10 (5–11)	0.48^§^
Central sensitisation (CSI > 40), %	**35%**	**60%**	**0.03**^**‡**^	39%	41%	0.87^‡^
Mental health score	76 (64–83)	72 (60–84)	0.52§	76 (68–84)	78 (57–83)	0.46^§^
Sleep quality (PSQI > 5), %	66	74	0.48^‡^	64	65	0.95^‡^
Physical activity (IPAQ)	10 (8–16)	10 (7–18)	0.90^§^	13 (8–18)	9.1 (7–16)	0.56^§^
Visceral adipose tissue (mm)	59 (22)	66 (26)	0.21^†^	59 (24)	50 (21)	0.24^†^
Placebo Q1 agree, %	91	94	0.57^‡^	92	94	0.75^‡^
Placebo Q2 agree, %	95	96	0.84^‡^	92	94	0.75^‡^
Placebo Q3 agree, %	93	87	0.34^‡^	92	88	0.69^‡^
Placebo Q4 agree, %	96	96	0.95^‡^	97	94	0.58^‡^

Values are presented as mean (SD) for normal distributed continuous data, as median with the interquartile range (25th–75th percentiles) for non-normal distributed continuous data and as percentages for categorical data. Sig. represents the p-value between the experimental intervention and control intervention or between those in the experimental group with a good outcome versus those with a poor outcome. ^†^Independent sample t-test ^‡^Pearson Chi square test; ^§^Independent sample Kruskall-Wallis; BMI, Body Mass Index; VAS, visual analogue scale (0–100); NDI, neck disability index (0–100); DASS21, depression, anxiety, stress score; MHI5, mental health inventory-5; IPAQ, international physical activity questionnaire presented in 1000 METs; mm, millimetre. Patients provided information if they agree with the following four statements. Q1: I believe this intervention will allow me to get better quicker; Q2: I believe this intervention will decrease my neck pain; Q3: I believe this intervention will make me more able to do the things I want to do; Q4: This seems like a logical way to treat neck pain. Modified from^48^. Bold represents a significant difference between groups (p < 0.05).

Finally, linear mixed models were used to further test whether the changes in pain scores in the experimental group were associated with the change in immediate neuroimmune responses. Linear regression analyses with an intention-to-treat approach were used to compare serum cortisol levels and differences in phenotypic analysis of peripheral white blood cells between the experimental group and control group, and between people with a good and poor outcome in the experimental group. Different models were tested: *Model 1*: differences in baseline values; *Model 2*: differences following the intervention; *Model 3*: differences following the intervention adjusted for baseline values; *Model 4*: Model 3 adjusted for estrogen medication use and menstrual cycle (only applicable for serum cortisol); *Model 5*: Model 3 adjusted for BMI, age and gender (only applicable for phenotyping of blood mononuclear cells) and *Model 6*: Model 3 adjusted for NDI score, PCS score, TSK score and CSI score (only applicable in the comparison experimental group versus control group). A p-value < 0.05 was considered statistically significant. Due to the exploratory nature of the study, p-values were not adjusted for multiple comparisons^29^.

### Informed consent

Informed consent has been obtained to publish in an online open-access publication.

## Results

### Participants

**Figure 3:**
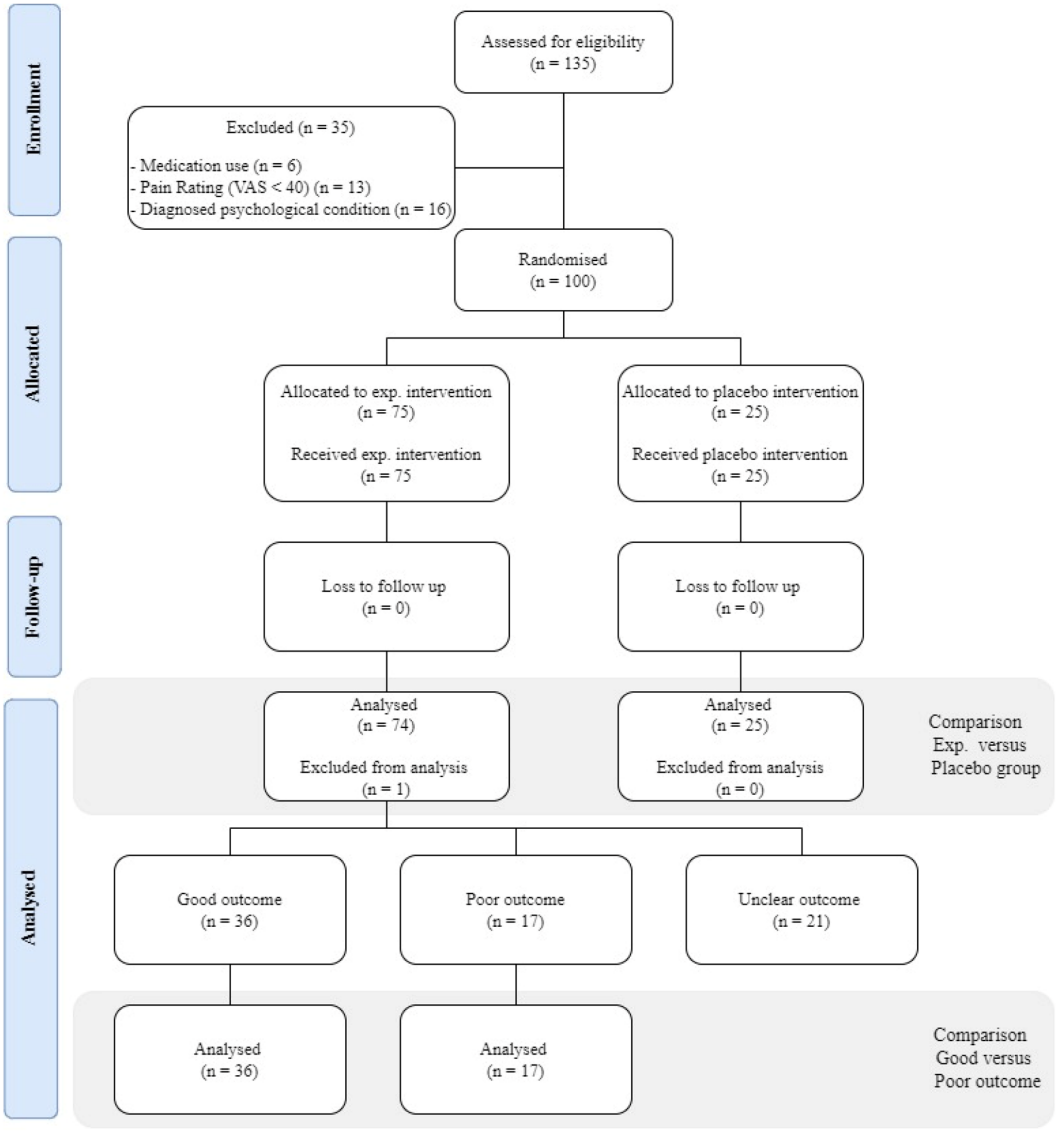
Flowchart of the study.

One hundred and thirty-five people with non-specific neck pain were assessed on the eligibility criteria, of whom 100 people met the selection criteria and agreed to participate in the study (Fig. 3). One participant in the experimental group had to be excluded due to a rheumatic disease diagnosis after data collection. At baseline, the control group experienced more disability, kinesiophobia, pain catastrophising, and central sensitisation compared to the experimental group (Table 2). Table 3 summarises the physical examination between group differences at baseline and follow- up. In the experimental group, 36 people (49%) met the criterion for a good outcome and 17 participants (22%) had a poor outcome. No differences at baseline were detected between those with a good outcome versus those with a poor outcome following the experimental intervention. Figure 3 provides the flow diagram of the study. There was no loss-to-follow up.

**Table 3:** Between group differences at baseline and follow-up.

	Time	Experimental vs. control		Good outcome vs. poor outcome	
		Mean differences (95%CI)	Sig.	Mean differences (95%CI)	Sig.
Pain intensity (VAS)	T0	− 5.0 (− 12.6, 2.6)	0.19	− 1.5 (− 9.4, 6.5)	0.72
	T1	**− 30.3 (− 38.2, − 22.6)**	**0.001**	**− 30.6 (− 36.8, − 24.5)**	**0.001**
	T2	**− 32.0 (− 40.4, − 23.6)**	**0.001**	**− 29.6 (− 36.6, − 22.6)**	**0.001**
PPT trapezius right	T0	− 9.9 (− 95.8, 76.0)	0.82	56.8 (− 56.1, 169.3)	0.32
	T1	8.6 (− 71.4, 88.6)	0.83	79.6 (− 24.1, 183.2)	0.13
	T2	4.7 (− 77.2, 86.6)	0.91	84.8 (− 20.3, 189.4)	0.11
PPT trapezius left	T0	6.9 (− 65.9, 79.8)	0.85	28.3 (− 80.0, 136.2)	0.61
	T1	47.1 (− 24.4, 118.1)	0.19	23.8 (− 75.7, 123.1)	0.63
	T2	63.3 (− 31.2, 157.4)	0.19	44.9 (− 55.3, 145.6)	0.37
PPT tibialis anterior right	T0	50.4 (− 65.9, 166.8)	0.39	28.3 (− 140.7, 197.2)	0.74
	T1	**116.2 (− 0.2, 233.3)**	**0.05**	78.9 (− 91.3, 249.7)	0.36
	T2	103.4 (− 16.3, 222.5)	0.09	54.8 (− 115.5, 224.5)	0.52
PPT sub occipitalis right	T0	7.5 (− 54.9, 69.9)	0.81	23.7 (− 64.0, 111.0)	0.59
	T1	43.8 (− 16.7, 104.5)	0.15	64.9 (− 21.5, 151.5)	0.14
	T2	49.6 (− 11.9, 111.8)	0.11	48.6 (− 38.8, 135.6)	0.27
PPT sub occipitalis left	T0	− 2.9 (− 62.4, 56.7)	0.93	20.6 (− 56.6, 97.8)	0.59
	T1	39.9 (− 16.3, 96.0)	0.16	38.6 (− 36.4, 113.7)	0.31
	T2	42.3 (− 16.1, 100.0)	0.15	20.2 (− 57.3, 97.7)	0.61
Cervical right rotation	T0	3.0 (− 13.1, 7.2)	0.56	**15.8 (28.3, 5.1)**	**0.005**
	T1	**16.9 (24.7, 9.2)**	**0.001**	**13.6 (21.4, 5.8)**	**0.001**
	T2	**22.4 (30.3, 14.5)**	**0.001**	**10.5 (17.4, 3.6)**	**0.003**
Cervical left rotation	T0	− 0.2 (− 7.4, 7.1)	0.96	1.8 (− 11.7, 8.0)	0.71
	T1	5.8 (− 0.2, 11.8)	0.06	1.4 (− 8.3, 5.5)	0.69
	T2	6.1 (− 0.2, 12.4)	0.06	3.6 (− 10.5, 3.3)	0.30
Temporal summation trapezius right^†^	T0	− 8.0 (− 20.8, 4.9)	0.22	− 1.8 (− 9.8, 6.2)	0.65
	T1	− 6.6 (− 19.1, 6.0)	0.30	− 1.0 (− 9.1, 7.1)	0.81
	T2	− 2.2 (− 14.4, 10.1)	0.73	− 1.0 (− 8.3, 6.3)	0.79
Temporal summation trapezius left^†^	T0	− 3.6 (− 18.4, 11.5)	0.64	− 4.81 (− 14.4, 4.8)	0.32
	T1	− 9.5 (− 24.0, 5.0)	0.19	− 2.9 (− 11.5, 5.8)	0.51
	T2	− 4.6 (− 19.2, 10.1)	0.53	− 0.7 (− 10.1, 8.8)	0.89
Temporal summation tib. ant.^†^	T0	− 14.7 (− 30.5, 1.0)	0.07	4.7 (− 7.2, 16.6)	0.42
	T1	**− 19.3 (− 35.5, − 3.1)**	**0.02**	8.1 (− 4.7, 20.8)	0.20
	T2	− 11.8 (− 26.2, 2.5)	0.10	0.4 (− 8.7, 9.4)	0.94
CROM-VAS right^†^	T1	**− 14.6 (− 21.2, − 8.1)**	**0.001**	3.4 (− 1.4, 6.8)	0.11
	T2	**− 20.3 (− 26.7, − 13.7)**	**0.001**	**− 5.6 (− 0.7, − 9.3)**	**0.01**
CROM-VAS left^†^	T1	**− 14.5 (− 20.7, − 08.2)**	**0.001**	**− 6.4 (− 2.1, − 9.7)**	**0.001**
	T2	**− 24.4 (− 31.3, − 17.5)**	**0.001**	**− 9.2 (− 4.1, − 13.1)**	**0.001**

Sig. represents the p-value between the experimental intervention and control intervention or between those in the experimental group with a good outcome versus those with a poor outcome. Linear mixed model analyses with fixed factor (time), covariate (group) and interaction (time * group) were used to assess differences between the groups at the three time moments. A random intercept was chosen to account for the correlated nature of multiple measurements from the same individual. ^†^Analysed using linear regression analysis based on T1 and T2 with T0-values as covariate. In the calculations for the wind-up phenomenon T0 zero-values were excluded from the analysis. Abbreviations: PPT: pressure pain threshold; ROM: range of motion; tib.ant.: tibiales anterior muscle; T0: baseline; T1: immediate follow-up; T2: 2 h follow-up. Bold represents a significant difference between groups (p < 0.05).

### Primary outcomes

**Figure 4:**
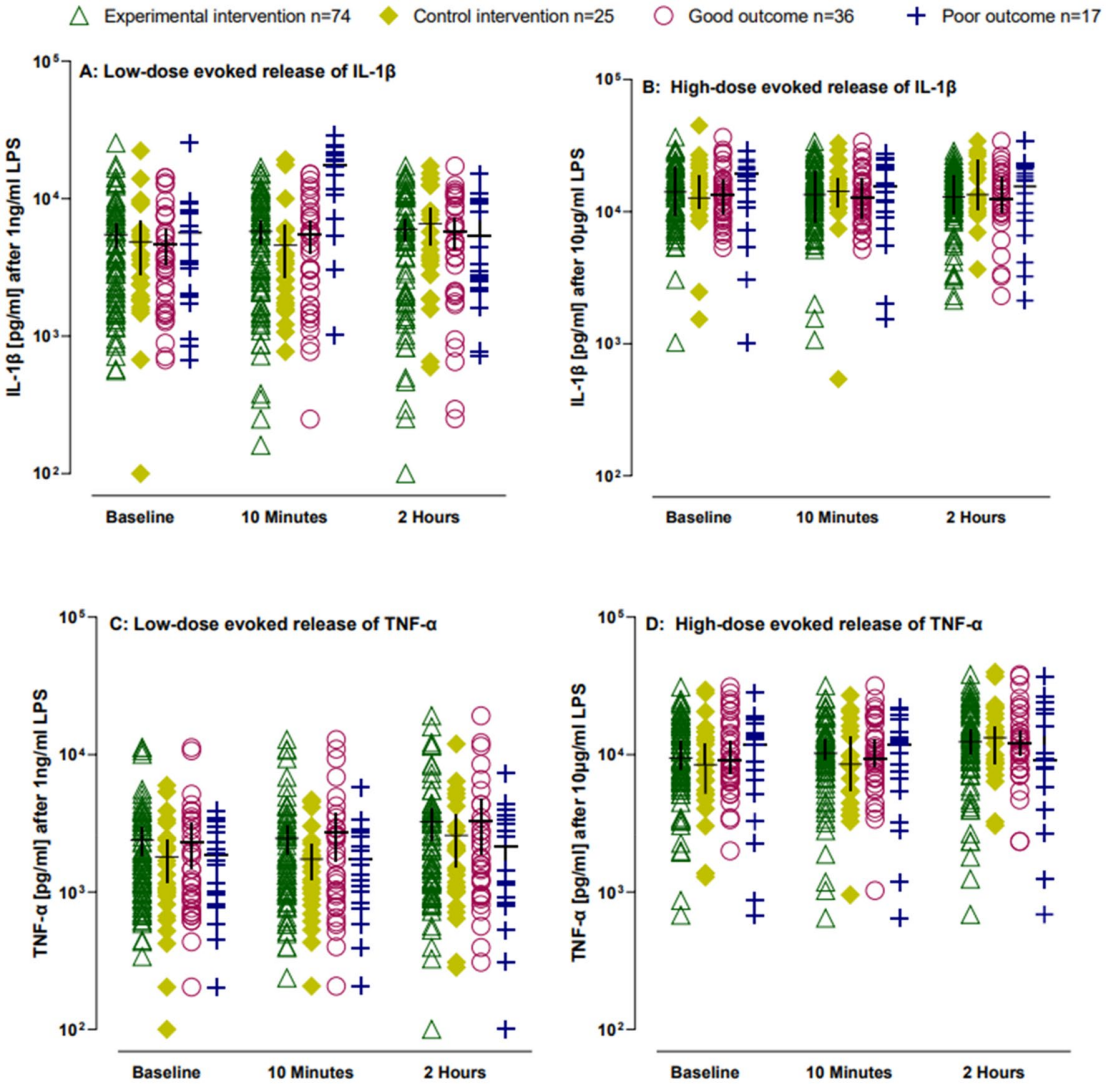
Immediate effects of the joint mobilisation/manipulation and placebo joint mobilisation/manipulation on the in vitro levels of IL-1β and TNF-α. Immediate effects of the control intervention, experimental intervention, of the experimental intervention in those having a good outcome and poor outcome on the in-vitro levels of IL-1β or TNF-α after whole blood stimulation with 1 ng/ml or 10 µg/ml LPS. (**A**) IL-1β levels following low-dose stimulation. (**B**) IL-1β levels following high stimulation. (**C**) TNF-α levels following low-dose stimulation. (**D**) TNF-α following high-dose stimulation. Lines represent median (*horizontal line*), and 25th–75th percentiles. TNF-α, tumor necrosis factor -α; IL-1β, interleukin -1β.

There were no significant or clinically meaningful differences in immediate whole blood evoked released in-vitro levels of IL-1β and TNF-α between the experimental group and control group. Between group differences (B-value) for low-dose stimulation 10 min following the interventions for IL-1β was 0.05 (95%CI − 0.09, 0.50) and 0.17 (95%CI − 0.16, 0.51) for TNF-α and at 2 h following the interventions the B-value for IL-1β was 0.26 (95%CI − 0.74, 0.21), and 0.11 (95%CI − 0.29, 0.50) for TNF-α. B-value for high-dose stimulation 10 min following the interventions for IL-1β was − 0.06 (95%CI − 0.35, 0.24) and 0.15 (95%CI − 0.19, 0.48) for TNF-α and the 2 h following the interventions B-value for IL-1β was − 0.25 (95%CI − 0.57, 0.06) and − 0.11 (95%CI − 0.48, 0.25) for TNF-α (Fig. 4). Normalising for monocyte count (Model 2) and adjusting for potential confounders (Model 3 and Model 4) did not change the results (Fig. 4).

### Secondary outcomes

#### Good versus poor outcome

No significant or clinically meaningful differences were detected in immediate neuroimmune responses between the experimental and control group, or between those with a good versus a poor outcome (Fig. 4, Appendix 2–9).

#### Associations between neuroimmune responses and pain reduction

We found no significant or meaningful associations between the immediate neuroimmune responses and pain reduction within the groups. Appendix 10 and 11 show the results of the association analyses.

#### Self-reported questionnaires and physical tests

**Figure 5:**
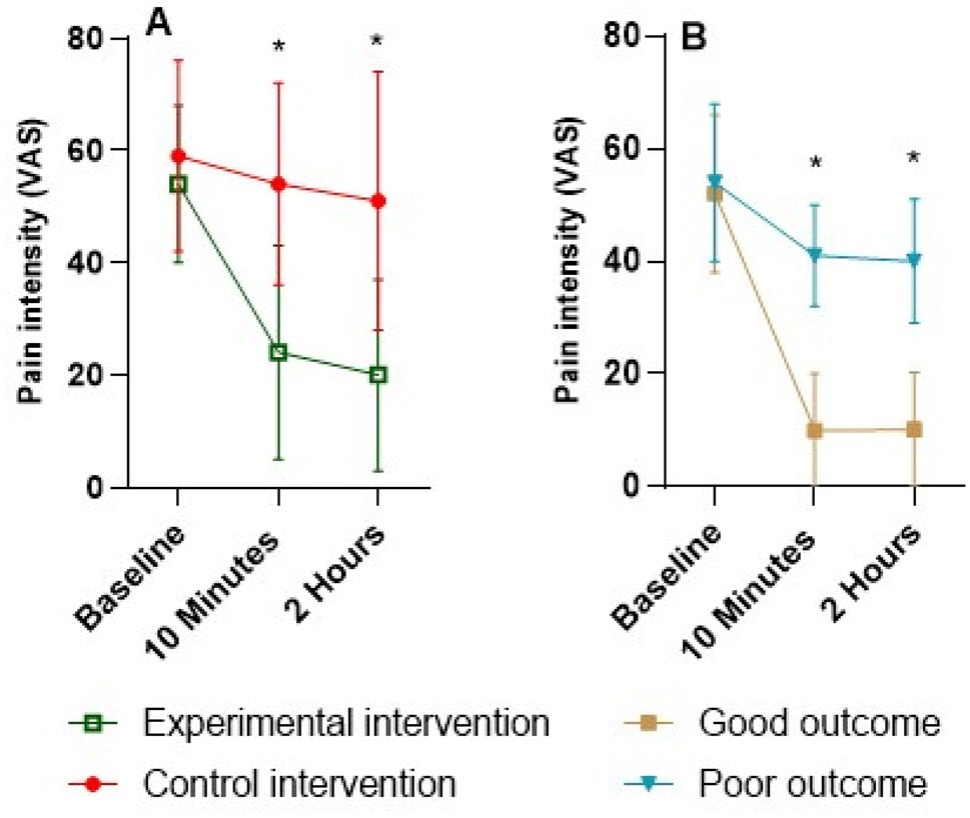
Pain intensity over time. (**A**) Pain intensity of the experimental intervention and control intervention at each time point. (**B**) Pain intensity of those in the experimental group classified as having a good outcome or poor outcome. T0 represents baseline, T1 immediately following the intervention, T2 2-h following the intervention. *Represents a significant difference between groups (p < 0.05).

People in the experimental group experienced a significant and clinically relevant reduction in pain intensity compared to the control group immediate after the intervention (mean (95%CI) between group difference in pain intensity (VAS) at 10 min: 30.3 mm (95%CI 38.2, 22.6), and at 2 h: 32.0 mm (95%CI 40.4, 23.6)) in favour of spinal mobilisation/manipulation (Fig. 5). The good outcome group experienced a significant and clinically relevant larger reduction in pain intensity compared to the poor outcome group immediately after the intervention (between group difference at 10 min: 30.6 mm (95%CI 36.8, 24.5), and at 2 h: 29.6 mm (95%CI − 36.6, − 22.6) in favour of the good outcome group (Fig. 5).

There were no meaningful differences in pressure pain thresholds between groups (p ≥ 0.11). There was a significant between groups difference for increase in cervical right rotation range of motion of 16.9° (95%CI 24.7, 9.21) at 10 min and 22.4° (95%CI 30.3, 14.5) at 2 h after the intervention in favour of the experimental group compared to the control group. The between group difference in reduction of temporal summation was 19 points (95%CI 35.5, 3.06) at 10 min after the intervention in favour of the experimental group compared to the control group, but at 2 h there was no significant difference between the groups. Significantly more people scored “slight improvements” in global perceived effect scores in the experimental group compared to the control group, and those with a good outcome compared to a poor outcome (Appendix 12).

No major or minor adverse events were reported.

## Discussion

In this study, we comprehensively evaluated the immediate systemic neuroimmune responses following spinal mobilisation/manipulation in people with non-specific neck pain. Although there were meaningful differences in the effect of spinal mobilisation/manipulation on clinical outcomes such as pain intensity and cervical range of motion, we did not identify differences in neuroimmune responses at a systemic level between the experimental and placebo intervention, nor between those with a good outcome versus a poor outcome in the experimental group, nor meaningful associations with pain reduction. Although we found some significant differences for the clinical outcomes, some findings may be related to type 1 errors due to multiple testing, suggesting some of the significant findings may be due to chance or random variation rather than a true effect. There is an increasing volume of literature regarding the effects of joint mobilisation/manipulation on neuroimmune responses in musculoskeletal conditions^4,6^. Despite the increased number of studies, high quality randomised clinical trials assessing neuroimmune responses in patients are limited^6^. Our results on inflammatory markers add to the current literature showing that joint mobilisation/manipulation has no immediate effect on the inflammatory markers studied at a systemic level. There are conflicting results in the literature on the potential to change serum/salivary cortisol following joint mobilisation/manipulation^30–32^. It is proposed that pain might be inhibited due to the anti-inflammatory/anti-nociceptive effects of cortisol. Our results do not support this hypothesis as we did not find meaningful differences between the experimental and control group, and those with a good versus a poor outcome in the experimental group, and could not detect significant associations between cortisol levels and pain reduction. The results of the previous studies^33–35^ have to be interpreted with caution as several factors may have confounded the serum cortisol levels, such as circadian rhythm, fasted state, medication use (anti-conceptive/estrogen), and the treatment instructions given to the patient^30,36^. In our study we controlled for all these factors which could probably explain the differences between our results and the previous studies.

Ex-vivo inflammatory markers are easy to measure, and therefore attractive to investigate in pain research^37,38^. Besides the advantages of ex-vivo inflammatory markers, several disadvantages need to be listed: first the cellular source of the ex-vivo inflammatory markers cannot be determined^39^; second, potential alterations in inflammatory marker concentrations dilutes into the systemic circulation; and third, temporal dynamics of inflammatory markers might be different per patient^40^. Therefore, in addition to the ex-vivo serum determination of inflammatory markers, whole blood in-vitro evoked release of inflammatory markers was used. In-vitro measurements have the advantage that the supernatants collected after stimulation contain all inflammatory substances preventing dilution into the systemic circulation^41^. However, the time between whole blood stimulation and collection of the supernatants has major effects on the immune response due to the temporal dynamics of the inflammatory response^42^. Our results are not in line with previous findings that joint mobilisation/manipulation affects in-vitro responsivity of whole blood cells to lipopolysaccharide (LPS)^12,15,16^. In contrast to the previous studies, we measured the in-vitro responsivity and ex-vivo inflammatory markers directly after joint mobilisation/manipulation in people with non-specific neck pain. This could partly explain the discrepancy found. One non-randomised controlled trial found that 4 weeks of treatment with joint manipulation resulted in a reduction of in-vitro responsivity in people with back pain^15^. However, in healthy participants, a single session of joint mobilisation/manipulation compared to a placebo mobilisation/manipulation showed an immediate time-dependent attenuation of LPS evoked release of inflammatory cytokine IL-1β and TNF-α^16^. Finally, a recent study in patients with low back pain showed that ^1^H-MRS increased choline levels in the thalamus, insula and somatosensory cortex following several sessions of spinal manipulation compared to sham intervention^43^. As ^1^H-MRS choline levels are linked to neuroinflammation^44^ these results might indicate central neuroimmune responses following joint manipulation. These results point to a compelling need to assess whether several treatment sessions in a clinical population, localised neuroimmune responses and a longer follow-up period might be needed to detect changes in neuroimmune responses following joint mobilisation/manipulation.

One important issue in determining the in-vitro responsivity of whole blood cells is to control for the number of cytokine producing cells and the number of cells cultured^42^. Because of the immunophenotyping performed in this study, we were able to normalise the in-vitro immune response for the number of monocytes. Notwithstanding, even after normalising for monocyte counts, no meaningful differences could be detected in immediate in-vitro neuroimmune responses. In contrast to the previous studies, our study is the first study which performed extensive phenotyping of peripheral blood mononuclear cells, and cell staining for activation marker HLA-DR and TLR-4 expression on monocytes in relation to joint mobilisation/manipulation. The negative finding of in-vitro whole blood stimulation is strengthened as we did not find differences in cell phenotyping and activation marker HLA-DR on monocytes and/or TLR-4 expression.

Several considerations should be noted when interpreting the findings. First, due to the non-linearity of the neuroimmune responses, Ln-transformation was necessary which makes the interpretation of the data more complicated. Second, we opted for unequal sample sizes. As we also wanted to compare people with a good versus poor outcome within the experimental group, the experimental group needed to be larger than the control group at baseline. However, we incorporated unequal group sizes in our a priori sample size estimation, thereby limiting a type 2 error. Thirdly, the effects of only a single sessions of joint mobilisation/manipulation on immediate neuroimmune responses were examined. This could seem to limit the clinical relevance but gives a better insight into the true effect of mobilisation/manipulation on neuroimmune outcomes by limiting the effects of contextual factors. Moreover, our aim was to understand the biological mechanisms behind pain reduction and not the clinical efficacy of mobilisation/manipulation. Also, a longer follow-up period might complicate establishing a cause-effect relationship between the intervention and systemic neuroimmune responses due to confounding factors which may introduce bias^18,19^. Not all participants in the control group were naïve to joint mobilisation/manipulations which might have affected the credibility of the placebo intervention^45^. Nonetheless, as we did not find statistical differences between the intervention expectations we believe this has only had a minor influence.

Finally, two minor protocol deviations are needed to be mentioned. First, we changed the label ‘short term effects’ to ‘immediate effects’ as we believe 2 h post intervention is better described still as ‘immediate’ rather than ‘short term’, which may reflect days or weeks. Secondly, due to an omission, pain intensity after two days was not recorded.

To conclude, we found that joint mobilisations/manipulation did not affect immediate systemic neuroimmune responses and no associations were found between neuroimmune responses and pain reduction. Having revealed that joint mobilisations/manipulation had no effect on systemic levels of inflammation, future research may focus on more localised neuroimmune responses (e.g., at the level of the dorsal root ganglion, spinal cord and brain^6^). Demonstrating changes in neuroimmune responses are more difficult in these locations, but these locations may show clearer changes. If demonstrated, it would show the complementary effects of joint mobilisation and manipulation and aerobic exercise on neuroimmune responses^6,46,47^.

### Supplementary Information

Supplementary Information.

## Data Availability

Individual deidentified participant data that underlie the results will be shared. Investigators whose proposed use of the data had been approved by an independent review committee identified for this purpose can access the data for individual participant data meta-analysis. Data will be available immediately upon publication. Proposals may be submitted up to 36 months following article publication. After 36 months the data will be available in our University’s data warehouse but without investigator support other than deposited metadata. Information regarding submitting proposals and accessing data may be found at https://research.vu.nl.
