# Supplementary material for: Immediate systemic neuroimmune responses following spinal mobilisation and manipulation in people with non-specific neck pain: a randomised placebo-controlled trial
Source: Sci Rep. 2023 Aug 7;13:12804. doi: 10.1038/s41598-023-39839-3 (PMC10406885; doi:10.1038/s41598-023-39839-3)
Supplement: Supplementary file 1 — Supplementary Information. [file 41598_2023_39839_MOESM1_ESM.docx]

**Appendix 1** Gating strategies for flow cytometry

**
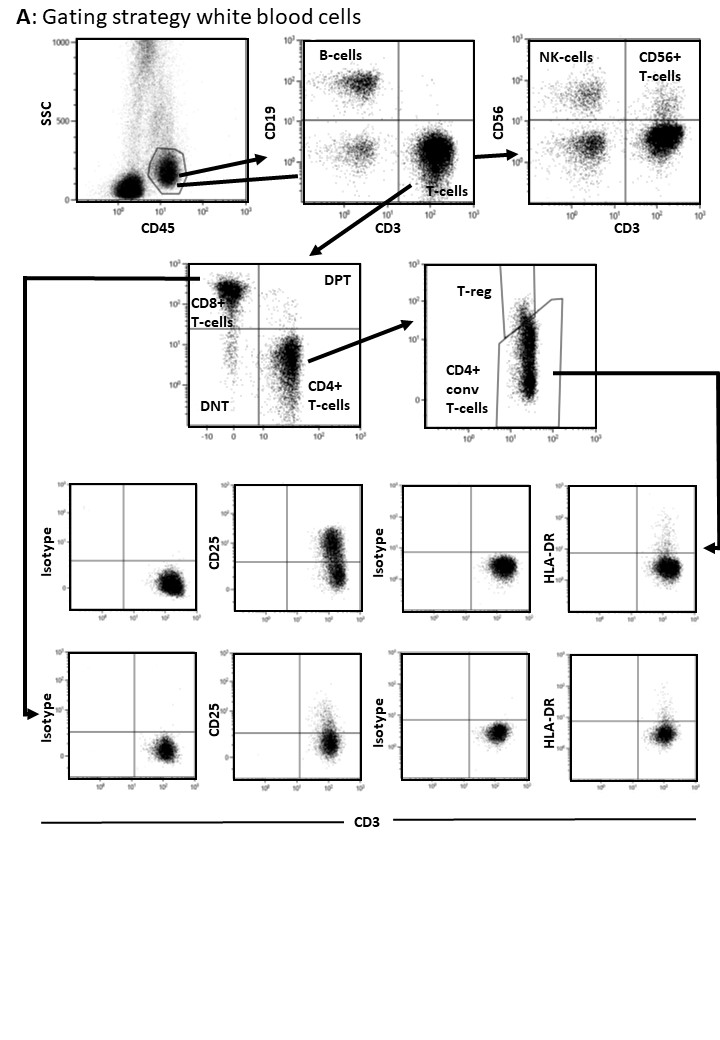

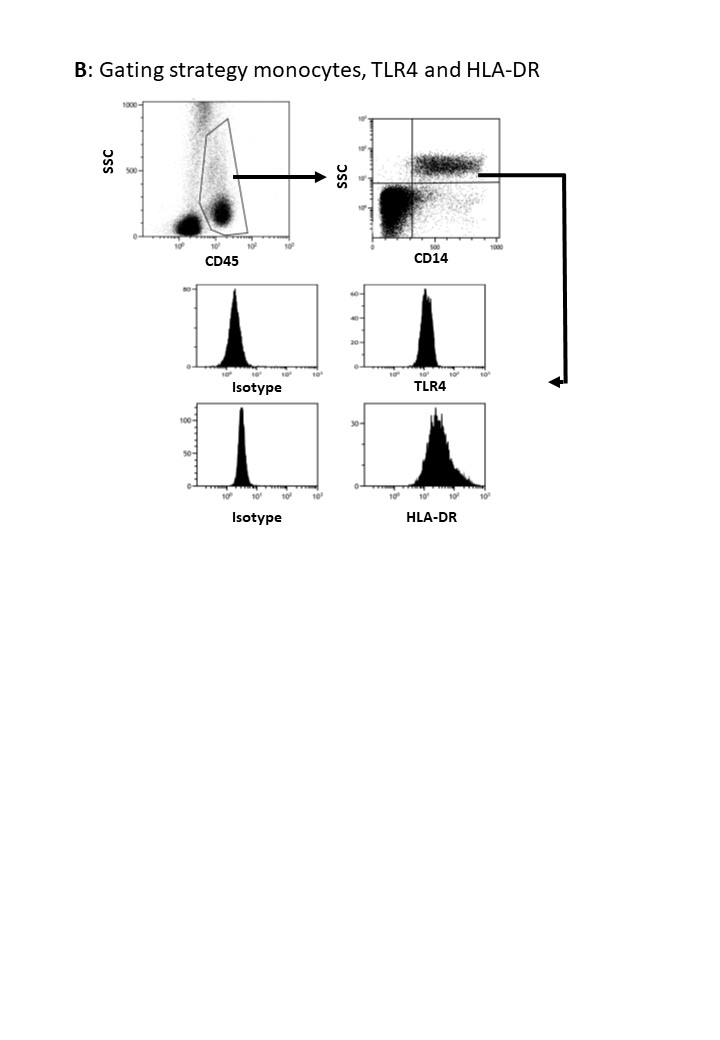
**

Panel **A:** Lymphocytes are gated based on low side scatter (SSC) and CD45 expression. Within the lymphocytes, CD19^+^ B-cells, CD3^+^ T-cells, CD56^+^CD3^-^ natural killer (NK) cells and CD56^+^ T-cells are gated. Within CD3^+^ T-cells CD4^+^CD8^-^ (CD4^+^ T-cells), CD4^-^CD8^+^ (CD8^+^ T-cells), CD4^+^CD8^+^ double positive T-cells (DPT) and CD4^-^ D8^-^ double negative T-cells (DNT) are gated. Within CD4^+^ T-cells, CD25hi T-regulatory (Treg) are gated, the remaining CD4^+^ T-cells are defined as conventional T-cells. CD25 and HLA-DR expression is analyzed on CD4^+^ conventional T-cells and CD8^+^ T-cells using appropriate isotype controls for gating. Panel **B:** Mononuclear cells (PBMC) are gated based on low/intermediate SSC and CD45 expression. Within the PBMC, CD14^+^ monocytes are gated. Activation markers TLR4 and HLA-DR expression on CD14^+^ monocytes is analysed and expression levels are corrected for background staining with appropriate isotype controls.

**Appendix 2** Differences in *ex-vivo* inflammatory markers between the experimental and control group.

|  | Time | B (95%CI:)  Model 1 | Sig. | Adjusted-B (95%CI:) model 2 | Sig. | Adjusted-B (95%CI:) model 3 | Sig. |
| --- | --- | --- | --- | --- | --- | --- | --- |
| hsCRP | T0 | 0.17 (-0.38, 0.72) | 0.54 | 0.42 (-0.08, 0.93) | 0.10 | -0.01 (-0.57, 0.56) | 0.99 |
|  | T1 | 0.17 (-0.38, 0.72) | 0.55 | 0.42 (-0.08, 0.93) | 0.10 | -0.01 (-0.57, 0.57) | 0.98 |
|  | T2 | 0.12 (-0.52, 0.75) | 0.72 | 0.36 (-0.24, 0.97) | 0.24 | -0.06 (-0.72, 0.59) | 0.84 |
| TNF-α | T0 | **0.28 (0.0, 0.54)** | **0.04** | **0.32 (0.05, 0.58)** | **0.02** | 0.25 (-0.03, 0.53)  ♂ 0.21 (-0.36, 0.77)  **♀ 0.36 (0.01, 0.69)** | 0.08  ♂ 0.46  **♀ 0.04** |
|  | T1 | 0.25 (-0.01, 0.51) | 0.06 | **0.29 (0.03, 0.55)** | **0.03** | 0.22 (-0.06, 0.50)  ♂ 0.22 (-0.33, 0.79)  ♀ 0.30 (-0.03, 0.64) | 0.12  ♂ 0.42  ♀ 0.07 |
|  | T2 | 0.17 (-0.11, 0.44) | 0.22 | 0.20 (-0.07, 0.48) | 0.14 | 0.14 (-0.15, 0.43)  ♂ 0.14 (-0.44, 0.71)  ♀ 0.22 (-0.13, 0.57) | 0.36  ♂ 0.63  ♀ 0.21 |
| TNF-RII | T0 | 0.04 (-0.14, 0.23) | 0.66 | 0.06 (-0.13, 0.25) | 0.53 | 0.05 (-0.14, 0.25) | 0.59 |
|  | T1 | -0.22 (-0.53, 0.09) | 0.17 | -0.21 (-0.52, 0.10) | 0.18 | -0.22 (-0.53, 0.10) | 0.17 |
|  | T2 | 0.04 (-0.14, 0.23) | 0.66 | 0.06 (-0.13, 0.24) | 0.54 | 0.05 (-0.14, 0.25) | 0.59 |
| IL-1β | T0 | 0.08 (-0.42, 0.58) | 0.76 | 0.09 (-0.41, 0.60) | 0.70 | 0.04 (-0.47, 0.55) | 0.89 |
|  | T1 | -0.05 (-0.62, 0.52) | 0.86 | -0.01 (-0.57, 0.57) | 0.99 | -0.06 (-0.65, 0.52) | 0.84 |
|  | T2 | -0.24 (-0.65, 0.15) | 0.22 | -0.20 (-0.61, 0.21) | 0.32 | -0.26 (-0.68, 0.16) | 0.22 |
| IL-1RA | T0 | 0.09 (-0.16, 0.33)  ♂0.03 (-0.33, 0.39)  ♀ 0.14 (-0.17, 0.46) | 0.49  ♂ 0.89  ♀ 0.37 | 0.22 (-0.01, 0.43) | 0.06 | 0.11 (-0.15, 0.36)  ♂ 0.07 (-0.27, 0.45)  ♀ 0.13 (-0.24, 0.51) | 0.41  ♂ 0.67  ♀ 0.51 |
|  | T1 | -0.01 (-0.24, 0.22)  ♂-0.04 (-0.37, 0.28)  ♀ 0.05 (-0.24, 0.34) | 0.93  ♂ 0.79  ♀ 0.73 | 0.12, (-0.07, 0.32) | 0.22 | 0.02 (-0.22, 0.26)  ♂ -0.01 (-0.30, 0.32)  ♀ 0.03 (-0.33, 0.39) | 0.89  ♂ 0.96  ♀ 0.87 |
|  | T2 | -0.01 (-0.24, 0.22)  ♂-0.05 (-0.39, 0.28)  ♀ 0.04 (-0.26, 0.34) | 0.93  ♂ 0.76  ♀ 0.78 | 0.11 (-0.08, 0.32) | 0.26 | 0.01 (-0.23, 0.25)  ♂ -0.01 (-0.33, 0.33)  ♀ 0.02 (-0.34, 0.38) | 0.94  ♂ 0.99  ♀ 0.92 |
| Inflammatory index | T0 | 0.25 (-0.09, 0.61) | 0.15 | **0.37 (0.03, 0.71)** | **0.03** | 0.24 (-0.13, 0.60) | 0.19 |
|  | T1 | -0.06 (-0.47, 0.35) | 0.76 | 0.04 (-0.37, 0.44) | 0.85 | -0.09 (-0.52, 0.34) | 0.67 |
|  | T2 | 0.09 (-0.25, 0.43) | 0.59 | 0.21 (-0.12, 0.53) | 0.23 | 0.07 (-0.28, 0.43) | 0.69 |
| Pro-inflammatory index | T0 | 0.29 (-0.03, 0.61) | 0.07 | **0.39 (0.08, 0.71)** | **0.01** | 0.23 (-0.11, 0.57) | 0.18 |
|  | T1 | 0.23 (-0.10, 0.55) | 0.18 | **0.34 (0.02, 0.65)** | **0.04** | 0.17 (-0.17, 0.52) | 0.32 |
|  | T2 | 0.09 (-0.23, 0.42) | 0.57 | 0.20 (-0.12, 0.53) | 0.22 | 0.04 (-0.31, 0.38) | 0.84 |
| Anti-inflammatory index | T0 | 0.19 (0.41, 0.77) | 0.53 | 0.36, (-0.22, 0.94) | 0.23 | 0.23 (-0.38, 0.85) | 0.45 |
|  | T1 | -0.55 (-1.41, 0.30) | 0.20 | -0.43 (-1.27, 0.42) | 0.32 | -0.54 (-1.42, 0.33) | 0.22 |
|  | T2 | 0.09 (-0.49, 0.67) | 0.76 | 0.26 (-0.31, 0.82) | 0.38 | 0.14 (-0.47, 0.74) | 0.66 |
| Ratio pro/anti inflammatory index | T0 | 0.88 (-2.86, 4.63) | 0.64 | 0.70 (-3.08, 4.48) | 0.71 | 0.75 (-3.05, 4.56) | 0.69 |
|  | T1 | 1.06 (-1.31, 3.44) | 0.38 | 0.83 (-1.55, 3.22) | 0.49 | 0.94 (-1.52, 3.42) | 0.45 |
|  | T2 | -0.88 (-2.98, 1.22) | 0.41 | -1.08 (-3.22, 1.05) | 0.32 | -1.02 (-3.24, 1.19) | 0.36 |

*Ex-vivo­* inflammatory markers were Ln-transformed. Sig. represents the p-value between the experimental intervention and control intervention. Mixed model analyses with fixed factor (time), covariate (group) and interaction (time*group) were used to detect differences between the groups at the three time moments. A random intercept was chosen to account for the correlated nature of multiple measurements from the same individual. T0: baseline; T1: immediate follow-up; T2: 2hours follow-up. Model 1: adjusted for plate number. Model 2: Additional adjusted for age; gender; body mass index. Model 3: Adjusted for plate number, NDI score, PCS total score, kinesiophobia and the presence of central sensitisation. ♂ effect modification for males; ♀ effect modification for females.

**Appendix 3** Differences in *ex-vivo* cortisol between the experimental and control group.

|  | B (95%CI:)  Model 1 | Sig. | B (95%CI:)  Model 2 | Sig. | Adjusted-B (95%CI:) Model 3 | Sig. | Adjusted-B (95%CI:) Model 4 | Sig. | Adjusted-B (95%CI:) Model 5 | Sig. |
| --- | --- | --- | --- | --- | --- | --- | --- | --- | --- | --- |
| Serum Cortisol | 0.12 (-0.07, 0.29) | 0.22 | 0.02 (-0.17, 0.20) | 0.85 | -0.02 (-0.07, 0.03) | 0.36 | -0.02 (-0.06, 0.03) | 0.49 | -0.02 (-0.06, 0.03) | 0.46 |

*Ex-vivo* cortisol was Ln-transformed. Sig. represents the p-value between the experimental intervention and control intervention.

Linear regression analysis was used to detect differences between the experimental and control intervention. Different models were tested: model 1: differences in baseline values; model 2: differences following the intervention values; model 3: differences following the intervention adjusted for baseline values; model 4: differences following the intervention adjusted for baseline values, estrogen medication use and menstrual cycle; and the final model 5: differences following the intervention values and adjusted for baseline, NDI score, PCS total score, kinesiophobia and the presence of central sensitisation.

**Appendix 4** Differences in inflammatory concentration after *in-vitro* stimulation of whole blood cells between the experimental and control group.

|  | Time | Stimulation | B (95%CI:)  Model 1 | Sig. | Norm. B (95%CI:)  Model 2 | Sig. | Norm. adjusted-B  (95%CI:) Model 3 | Sig. | Norm. adjusted-B  (95%CI:) Model 4 | Sig. |
| --- | --- | --- | --- | --- | --- | --- | --- | --- | --- | --- |
| TNF-α | T0 | 1ng/ml | 0.23 (-0.14, 0.60)  ♂ 0.09 (-0.69, 0.89)  ♀ 0.19 (-0.21, 0.59) | 0.23  ♂ 0.80  ♀ 0.36 | 0.21 (-0.18, 0.62) | 0.29 | 0.24 (-0.16, 0.64) | 0.24 | 0.20 (-0.22, 0.62) | 0.35 |
|  |  | 10µg/ml | 0.16 (-0.18, 0.51)  ♂ -0.01 (-0.55, 0.54)  ♀ 0.16 (-0.25, 0.56) | 0.35  ♂ 0.97  ♀ 0.45 | 0.14 (-0.25, 0.54) | 0.46 | 0.19 (-0.19, 0.59) | 0.32 | 0.12 (-0.30, 0.54) | 0.58 |
|  | T1 | 1ng/ml | 0.17 (-0.16, 0.51)  ♂ -0.07 (-0.68, 0.53)  ♀ 0.17 (-0.21, 0.55) | 0.31  ♂ 0.81  ♀ 0.38 | 0.11 (-0.23, 0.46) | 0.50 | 0.15 (-0.19, 0.51) | 0.39 | 0.11 (-0.26, 0.48) | 0.56 |
|  |  | 10µg/ml | 0.15 (-0.19, 0.48)  ♂ -0.01 (-0.62, 0.62)  ♀ 0.15 (-0.23, 0.53) | 0.38  ♂ 0.99  ♀ 0.45 | 0.13 (-0.25, 0.51) | 0.47 | 0.19 (-0.17, 0.56) | 0.29 | 0.11 (-0.28, 0.51) | 0.58 |
|  | T2 | 1ng/ml | 0.11 (-0.29, 0.50)  ♂ -0.36 (-1.24, 0.49)  ♀ 0.20 (-0.23, 0.63) | 0.61  ♂ 0.39  ♀ 0.37 | 0.06 (-0.34, 0.46) | 0.77 | 0.09 (-0.31, 0.50) | 0.64 | 0.06 (-0.36, 0.47) | 0.79 |
|  |  | 10µg/ml | -0.11 (-0.48, 0.25)  ♂ -0.38 (-1.07, 0.31)  ♀ -0.06 (-0.48, 0.36) | 0.55  ♂ 0.27  ♀ 0.77 | -0.13 (-0.51, 0.25) | 0.49 | -0.07 (-0.45, 0.31) | 0.70 | -0.16 (-0.56, 0.25) | 0.45 |
| IL-1β | T0 | 1ng/ml | 0.09 (-0.34, 0.54) | 0.66 | 0.09 (-0.37, 0.55) | 0.71 | 0.13 (-0.33, 0.60) | 0.57 | 0.07 (-0.42, 0.56) | 0.79 |
|  |  | 10µg/ml | 0.0-5 (-0.22, 0.33) | 0.70 | 0.04 (-0.30, 0.37) | 0.68 | 0.09 (-0.25, 0.43) | 0.61 | 0.03 (-0.33, 0.39) | 0.86 |
|  | T1 | 1ng/ml | 0.09 (-0.32, 0.50) | 0.66 | 0.04 (0.41, 0.48) | 0.86 | 0.10 (-0.34, 0.55) | 0.65 | 0.03 (-0.45, 0.50) | 0.89 |
|  |  | 10µg/ml | -0.06 (-0.35, 0.24) | 0.71 | -0.08 (-0.42, 0.27) | 0.67 | -0.01 (-0.36, 0.33) | 0.95 | -0.07 (-0.43, 0.29) | 0.71 |
|  | T2 | 1ng/ml | -0.26 (-0.74, 0.21) | 0.26 | -0.31 (-0.79, 0.17) | 0.20 | -0.25 (-0.73, 0.23) | 0.31 | -0.32 (-0.82, 0.18) | 0.21 |
|  |  | 10µg/ml | -0.25 (-0.57, 0.06) | 0.12 | -0.28 (-0.61, 0.05) | 0.10 | -0.22 (-0.56, 0.11) | 0.19 | -0.28 (-0.63, 0.07) | 0.11 |
| IL-1RA | T0 | 1ng/ml | 0.06 (-0.17, 0.29) | 0.63 | 0.05 (-0.25, 0.34) | 0.76 | 0.15 (-0.14, 0.43) | 0.32 | 0.06 (-0.24, 0.37) | 0.69 |
|  |  | 10µg/ml | -0.07 (-0.24, 0.09) | 0.40 | -0.09 (-0.35, 0.18)  ♂0.13 (-0.35, 0.61)  ♀ -0.15 (-0.46, 0.16) | 0.52  ♂ 0.59  ♀ 0.33 | 0.01 (-0.26, 0.26) | 0.98 | -0.07 (-0.35, 0.20)  ♂ 0.06 (-0.41, 0.54)  ♀ -0.11 (-0.47, 0.25) | 0.59  ♂ 0.79  ♀ 0.53 |
|  | T1 | 1ng/ml | -0.09 (-0.26, 0.07) | 0.28 | -0.13 (-0.36, 0.10) | 0.28 | -0.02 (-0.23, 0.19) | 0.87 | -0.11 (-0.35, 0.14) | 0.38 |
|  |  | 10µg/ml | -0.07 (-0.24, 0.08) | 0.36 | -0.10 (-0.32, 0.11)  ♂ 0.11 (-0.28, 0.52)  ♀ -0.18 (-0.43, 0.08) | 0.35  ♂ 0.52  ♀ 0.17 | -0.01 (-0.21, 0.19) | 0.94 | -0.09 (-0.31, 0.14)  ♂ 0.05 (-0.34, 0.44)  ♀ -0.13 (-0.44, 0.18) | 0.45  ♂ 0.80  ♀ 0.39 |
|  | T2 | 1ng/ml | -0.12 (-0.36, 0.11) | 0.30 | -0.15 (-0.40, 0.10) | 0.24 | -0.04 (-0.28, 0.19) | 0.72 | -0.13 (-0.40, 0.13) | 0.32 |
|  |  | 10µg/ml | -0.09 (-0.28, 0.09) | 0.31 | -0.12 (-0.04, 0.09)  ♂ 0.01 (-0.40, 0.40)  ♀ -0.16 (-0.42, 0.10) | 0.28  ♂ 0.99  ♀ 0.23 | -0.03 (-0.24, 0.18) | 0.79 | -0.11 (-0.34, 0.13)  ♂ -0.06 (-0.46, 0.33)  ♀ -0.12 (-0.43, 0.20) | 0.36  ♂ 0.74  ♀ 0.46 |
| IL-10 | T0 | 1ng/ml | 0.25 (-0.11, 0.61) | 0.18 | 0.23 (-0.15, 0.62) | 0.24 | 0.24 (-0.14, 0.61) | 0.21 | 0.19 (-0.21, 0.59) | 0.35 |
|  |  | 10µg/ml | 0.29 (-0.03, 0.61) | 0.07 | 0.26 (-0.06, 0.57) | 0.10 | 0.22 (-0.07, 0.52) | 0.14 | 0.19 (-0.12, 0.49) | 0.24 |
|  | T1 | 1ng/ml | 0.11 (-0.23, 0.45) | 0.54 | 0.06 (-0.27, 0.39) | 0.71 | 0.08 (-0.24, 0.39) | 0.66 | 0.02 (-0.33, 0.36) | 0.93 |
|  |  | 10µg/ml | 0.27 (-0.07, 0.61) | 0.12 | 0.22 (-0.12, 0.57) | 0.10 | 0.18 (-0.14, 0.51) | 0.28 | 0.15 (-0.19, 0.49) | 0.39 |
|  | T2 | 1ng/ml | -0.01 (-0.36, 0.35) | 0.99 | -0.04 (-0.39, 0.31) | 0.82 | -0.01 (-0.33, 0.33) | 0.97 | -0.06 (-0.42, 0.29) | 0.73 |
|  |  | 10µg/ml | 0.10 (-0.21, 0.42) | 0.53 | 0.02 (-0.39, 0.44) | 0.92 | 0.01 (-0.38, 0.40) | 0.96 | -0.02 (-0.43, 0.38) | 0.91 |
| CCL2 | T0 | 1ng/ml | 0.21 (-0.09, 0.51) | 0.18 | 0.19 (-0.13, 0.53) | 0.25 | 0.20 (-0.14, 0.54) | 0.24 | 0.19 (-0.16, 0.54) | 0.29 |
|  |  | 10µg/ml | 0.05 (-0.26, 0.37) | 0.74 | 0.02 (-0.30, 0.34) | 0.92 | -0.01 (-0.32, 0.31)  ♂ -0.39 (-1.03, 0.23)  ♀ 0.15 (-0.22, 0.51) | 0.95  ♂ 0.21  ♀ 0.44 | 0.09 (-0.25, 0.41) | 0.61 |
|  | T1 | 1ng/ml | 0.16 (-0.11, 0.43) | 0.25 | 0.15 (-0.14, 0.43) | 0.32 | 0.16 (-0.13, 0.45) | 0.28 | 0.14 (-0.16, 0.44) | 0.35 |
|  |  | 10µg/ml | 0.09 (-0.23, 0.43) | 0.57 | 0.05 (-0.25, 0.35) | 0.76 | 0.03 (-0.27, 0.33)  ♂ -0.51 (-1.16, 0.15)  ♀ 0.23 (-0.11, 0.56) | 0.87  ♂ 0.12  ♀ 0.18 | 0.12 (-0.19, 0.44) | 0.45 |
|  | T2 | 1ng/ml | 0.05 (-0.27, 0.37) | 0.76 | 0.03 (-0.29, 0.35) | 0.86 | 0.04 (-0.28, 0.36) | 0.82 | 0.02 (-0.31, 0.36) | 0.89 |
|  |  | 10µg/ml | 0.14 (-0.20, 0.48) | 0.42 | 0.09 (-0.22, 0.41) | 0.56 | 0.07 (-0.25, 0.38)  ♂ -0.36 (-1.05, 0.17)  ♀ 0.23 (-0.12, 0.57) | 0.66  ♂ 0.28  ♀ 0.19 | 0.16 (-0.16, 0.49) | 0.32 |
| CCL3 | T0 | 1ng/ml | **0.44 (0.02, 0.86)** | **0.04** | **0.43 (-0.01, 0.86)** | **0.05** | **0.45 (0.01 (0.90)** | **0.04** | 0.34 (-0.11, 0.79) | 0.14 |
|  |  | 10µg/ml | 0.17 (-0.07, 0.41)  ♂ -0.01 (-0.43, 0.41)  ♀ 0.21 (-0.05, 0.49) | 0.15  ♂ 0.96  ♀ 0.11 | 0.17 (-0.14, 0.47) | 0.27 | 0.21 (-0.09, 0.51) | 0.19 | 0.09 (-0.22, 0.42) | 0.54 |
|  | T1 | 1ng/ml | 0.20 (-0.10, 0.51) | 0.19 | 0.15 (-0.16, 0.47) | 0.34 | 0.19 (-0.13, 0.51) | 0.25 | 0.07 (-0.26, 0.41) | 0.67 |
|  |  | 10µg/ml | 0.11 (-0.13, 0.35)  ♂ -0.07 (-0.58, 0.43)  ♀ 0.16 (-0.09, 0.42) | 0.37  ♂ 0.77  ♀ 0.21 | 0.11 (-0.17, 0.38) | 0.46 | 0.15 (-0.13, 0.43) | 0.30 | 0.04 (-0.26, 0.33) | 0.81 |
|  | T2 | 1ng/ml | 0.05 (-0.39, 0.49) | 0.84 | 0.01 (-0.43, 0.44) | 0.99 | 0.04 (-0.41, 0.48) | 0.88 | -0.08 (-0.54, 0.37) | 0.72 |
|  |  | 10µg/ml | - 1. (-0.26, 0.28)   ♂ -0.29 (-0.79, 0.19)  ♀ 0.12 (-0.19, 0.44) | 0.95  ♂ 0.24  ♀ 0.43 | -0.01 (-0.29, 0.28) | 0.98 | 0.03 (-0.25, 0.33) | 0.81 | -0.07 (-0.37, 0.23) | 0.63 |
| CCL4 | T0 | 1ng/ml | 0.30 (-0.02, 0.63) | 0.07 | 0.06 (-0.20, 0.31) | 0.67 | 0.08 (-0.18, 0.35) | 0.56 | -0.01 (-0.27, 0.27) | 0.99 |
|  |  | 10µg/ml | 0.11 (-0.05, 0.26)  ♂ -0.13 (-0.37, 0.13)  ♀ 0.17 (-0.07, 0.34) | 0.21  ♂ 0.32  ♀ 0.96 | 0.09 (-0.17, 0.34)  ♂ 0.06 (-0.37, 0.49)  ♀ 0.08 (-0.25, 0.41) | 0.51  ♂ 0.79  ♀ 0.62 | 0.12 (-0.14, 0.38) | 0.36 | 0.04 (-0.23, 0.32) | 0.76 |
|  | T1 | 1ng/ml | 0.08 (-0.14, 0.31) | 0.48 | 0.05 (-0.19, 0.29) | 0.67 | 0.08 (-0.17, 0.33) | 0.53 | -0.01 (-0.26, 0.26) | 0.97 |
|  |  | 10µg/ml | 0.04 (-0.12, 0.21)  ♂ -0.18 (-0.48, 0.11)  ♀ 0.10 (-0.08, 0.29) | 0.62  ♂ 0.22  ♀ 0.28 | 0.02 (-0.19, 0.23)  ♂ -0.02 (-0.41, 0.36)  ♀ 0.01 (-0.24, 0.28) | 0.86  ♂ 0.91  ♀ 0.91 | 0.06 (-0.16, 0.28) | 0.59 | -0.02 (-0.25, 0.21) | 0.85 |
|  | T2 | 1ng/ml | -0.02 (-0.36, 0.32) | 0.98 | -0.05 (-0.37, 0.26) | 0.76 | -0.02 (-0.34, 0.29) | 0.87 | -0.11 (-0.43, 0.22) | 0.51 |
|  |  | 10µg/ml | - 1. (-0.15, 0.19)   ♂ -0.31 (-0.57, -0.04)  ♀ 0.13 (-0.07, 0.34) | 0.79  ♂ 0.03  ♀ 0.20 | -0.01 (-0.21, 0.21)  ♂ -0.14 (-0.49, 0.20)  ♀ 0.04 (-0.23, 0.32) | 0.99  ♂ 0.41  ♀ 0.76 | 0.04 (-0.17, 0.25) | 0.72 | -0.04 (-0.27, 0.18) | 0.69 |
| Inflammatory index | T0 | 1ng/ml | 0.22 (-0.04, 0.49)  ♂ 0.14 (-0.51, 0.81)  ♀ 0.19 (-0.04, 0.44) | 0.10  ♂ 0.66  ♀ 0.10 | 0.17 (-0.29, 0.62) | 0.28 | 0.25 (-0.11, 0.62) | 0.18 | 0.16 (-0.22, 0.54) | 0.40 |
|  |  | 10µg/ml | 0.17 (-0.10, 0.45)  ♂ -0.17 (-0.65, 0.30)  ♀ 0.27 (-0.06, 0.59) | 0.21  ♂ 0.46  ♀ 0.11 | 0.16 (-0.29, 0.62) | 0.48 | 0.23 (-0.23, 0.69) | 0.32 | 0.11 (-0.37, 0.59) | 0.65 |
|  | T1 | 1ng/ml | 0.11 (-0.11, 0.32)  ♂ -0.19 (-0.62, 0.22)  ♀ 0.16 (-0.07, 0.39) | 0.35  ♂ 0.35  ♀ 0.17 | 0.08 (-0.31, 0.47) | 0.69 | 0.13 (-0.19, 0.46) | 0.42 | 0.04 (-0.30, 0.38) | 0.82 |
|  |  | 10µg/ml | 0.11 (-0.18, 0.41)  ♂ -0.19 (-0.76, 0.38)  ♀ 0.18 (-0.14, 0.50) | 0.45  ♂ 0.50  ♀ 0.27 | 0.07 (-0.31, 0.47) | 0.69 | 0.16 (-0.24, 0.55) | 0.44 | 0.03 (-0.38, 0.44) | 0.89 |
|  | T2 | 1ng/ml | -0.03 (-0.31, 0.25)  ♂ -0.51 (-1.21, 0.19)  ♀ 0.11 (-0.13, 0.35) | 0.84  ♂ 0.16  ♀ 0.37 | -0.11 )-0.48, 0.26) | 0.56 | -0.03 (-0.41, 0.36) | 0.88 | -0.12 (-0.52, 0.27) | 0.54 |
|  |  | 10µg/ml | -0.05 (-0.36, 0.26)  **♂ -0.58 (-1.15, -0.02)**  ♀ 0.13 (-0.23, 0.49) | 0.77  **♂ 0.04**  ♀ 0.48 | -0.11 (-0.48, 0.27) | 0.56 | -0.02 (-0.41, 0.35) | 0.89 | -0.16 (-0.56, 0.25) | 0.45 |
| Pro inflammatory index | T0 | 1ng/ml | 0.24 (-0.04, 0.50) | 0.09 | 0.18 (-0.29, 0.65)  ♂ 0.32 (-0.37, 1.03)  ♀ 0.09 (-0.37, 0.56) | 0.45  ♂ 0.35  ♀ 0.69 | 0.24 (-0.13, 0.63) | 0.20 | 0.16 (-0.23, 0.55) | 0.43 |
|  |  | 10µg/ml | 0.18 (-0.11, 0.47)  ♂ -0.22 (-0.72, 0.28)  ♀ 0.27 (-0.06, 0.60) | 0.22  ♂ 0.38  ♀ 0.11 | 0.18 (-0.29, 0.65) | 0.45 | 0.25 (-0.23, 0.73) | 0.31 | 0.13 (-0.37, 0.63) | 0.62 |
|  | T1 | 1ng/ml | 0.14 (-0.07, 0.35) | 0.19 | 0.08 (-0.33, 0.49)  ♂ -0.10 (-0.80, 0.59)  ♀ 0.13 (-0.24, 0.50) | 0.68  ♂ 0.77  ♀ 0.49 | 0.17 (-0.17, 0.51) | 0.33 | 0.07 (-0.29, 0.44) | 0.67 |
|  |  | 10µg/ml | 0.11 (-0.19, 0.41)  ♂ -0.28 (-0.88, 0.32)  ♀ 0.19 (-0.12, 0.51) | 0.49  ♂ 0.34  ♀ 0.23 | 0.08 (-0.32, 0.49) | 0.69 | 0.16 (-0.25, 0.57) | 0.44 | 0.04 (-0.39, 0.48) | 0.86 |
|  | T2 | 1ng/ml | -0.01 (-0.29, 0.27) | 0.94 | -0.09 (-0.49, 0.29)  ♂ -0.61 (-1.71, 0.48)  ♀ 0.08 (-0.29, 0.45) | 0.63  ♂ 0.27  ♀ 0.67 | -0.03 (-0.45, 0.39) | 0.90 | -0.12 (-0.55, 0.32) | 0.59 |
|  |  | 10µg/ml | -0.05 (-0.37, 0.27)  ♂ -0.65 (-1.23, -0.07)  ♀ 0.14 (-0.23, 0.51) | 0.76  ♂ 0.03  ♀ 0.46 | -0.10 (-0.49, 0.29) | 0.63 | -0.02 (-0.42, 0.37) | 0.91 | -0.15 (-0.57, 0.27) | 0.49 |
| Anti-inflammatory index | T0 | 1ng/ml | 0.20 (-0.11, 0.51) | 0.21 | 0.14 (-0.33, 0.61) | 0.56 | 0.27 (-0.13, 0.67) | 0.19 | 0.18 (-0.25, 0.61) | 0.40 |
|  |  | 10µg/ml | 0.17 (-0.21, 0.54) | 0.38 | 0.14 (-0.33, 0.62) | 0.56 | 0.21 (-0.26, 0.67) | 0.39 | 0.09 (-0.41, 0.59) | 0.73 |
|  | T1 | 1ng/ml | 0.02 (-0.26, 0.29) | 0.90 | 0.08 (-0.36, 0.53) | 0.71 | 0.05 (-0.28, 0.37) | 0.78 | -0.05 (-0.40, 0.31) | 0.77 |
|  |  | 10µg/ml | 0.14 (-0.25, 0.53) | 0.49 | 0.08 (-0.36, 0.53) | 0.71 | 0.15 (-0.29, 0.59) | 0.51 | 0.03 (-0.44, 0.50) | 0.89 |
|  | T2 | 1ng/ml | -0.07 (-0.38, 0.24) | 0.65 | -0.13 (-0.61, 0.35) | 0.61 | -0.03 (-0.37, 0.32) | 0.86 | -0.13 (-0.49, 0.24) | 0.49 |
|  |  | 10µg/ml | -0.03 (-0.43, 0.37) | 0.89 | -0.13 (-0.60, 0.35) | 0.60 | -0.03 (-0.50, 0.12) | 0.19 | -0.16 (-0.65, 0.34) | 0.54 |
| Ratio pro/anti inflammatory index | T0 | 1ng/ml | 1.71 (-1.95, 5.37) | 0.36 | -0.25 (-2.12, 1.62) | 0.79 | -0.22 (-2.13, 1.67)  ♂ -1.55 (-5.68, 2.57)  ♀ 0.21 (-1.78, 2.22) | 0.81  ♂ 0.45  ♀ 0.83 | -0.63 (-2.56, 1.31) | 0.52 |
|  |  | 10µg/ml | -2.97 (-7.68, 1.73) | 0.21 | **1.62 (0.47, 2.76)** | **<0.01** | **1.75 (0.61), 2.90)** | **0.003** | **1.96 (0.77, 3.15)** | **<0.01** |
|  | T1 | 1ng/ml | 0.09 (-2.47, 2.66) | 0.94 | -1.58 (-3.44, 0.27) | 0.09 | -1.53 (-3.43, 0.37)  ♂ -4.31 (-9.52, 0.88)  ♀ -0.70 (-2.21, 0.78) | 0.11  ♂ 0.10  ♀ 0.35 | **-1.92 (-3.87, 0.03)** | **0.05** |
|  |  | 10µg/ml | -0.05 (-1.96, 1.87) | 0.96 | 4.32 (-0.77, 9.43) | 0.10 | 4.47 (-0.66, 9.61) | 0.09 | 4.66 (-0.46, 9.79) | 0.07 |
|  | T2 | 1ng/ml | -1.43 (-4.94, 2.08) | 0.42 | -0.02 (-17.5, 17.5) | 0.99 | 0.11 (-17.6, 17.7)  ♂ -0.54 (-3.86, 2.77)  ♀ 0.92 (-24.8, 26.7) | 0.99  ♂ 0.21  ♀ 0.94 | -0.28 (-17.9, 17.4) | 0.98 |
|  |  | 10µg/ml | 2.84 (-9.33, 15.0) | 0.64 | -0.06 (-2.22, 0.15) | 0.72 | 0.08 (-2.29, 2.45) | 0.95 | 0.26 (-2.11, 2.65) | 0.83 |

*In-vitro­* inflammatory markers were Ln-transformed. Sig. represents the p-value between the experimental intervention and control intervention. Mixed model analyses with fixed factor (time), covariate (group) and interaction (time*group) were used to detect differences between the groups at the three time moments. A random intercept was chosen to account for the correlated nature of multiple measurements from the same individual. T0: baseline; T1: immediate follow-up; T2: 2hours follow-up. Model 1: adjusted for plate number. Model 2: additional normalised /1000 monocytes. Model 3: Additional adjusted for age; gender; body mass index. Model 4: Adjusted for plate number, NDI score, PCS total score, kinesiophobia and the presence of central sensitisation. ♂ effect modification for males; ♀ effect modification for females.

**Appendix 5** Differences in cell staining and blood phenotyping between the experimental and control group.

| PBMC Phenotype | B (95%CI:) model 1 | Sig. | B (95%CI:) model 2 | Sig. | B (95%CI:) model 3 | Sig. | B (95%CI:) model 4 | Sig. | B (95%CI:) model 5 | Sig. |
| --- | --- | --- | --- | --- | --- | --- | --- | --- | --- | --- |
| Leucocytes^1^ | 0.16 (-0.74, 1.06) | 0.73 | 0.26 (-0.76, 1.28) | 0.62 | 0.07 (-0.42, 0.56) | 0.78 | -0.01 (-0.51, 0.49) | 0.99 | 0.15 (-0.41, 0.71) | 0.59 |
| PBMC^1^ | 29.2 (-414.4, 473.4) | 0.89 | -27.4 (-409.3, 355.3) | 0.89 | -53.0 (-273.5, 167.4) | 0.63 | -94.1 (-310.3, 122.3) | 0.39 | -78.2 (-316.2, 159.2) | 0.52 |
| Lymfocytes^1^ | 12.6 (-400.3, 425.3) | 0.95 | -32.1 (-388.3, 323.2) | 0.86 | -47.0 (-243.2, 149.4) | 0.64 | -73.7 (-266.3, 118.3) | 0.45 | -75.8 (-287.3, 135.3) | 0.48 |
| Monocytes (scatter)^1^ | 17.7 (-35.9, 71.3)  ♂ -62.3 (-164.2, 40.3)  ♀ 44.8 (-15.1, 104.2) | 0.51  ♂ 0.23  ♀ 0.14 | -3.5 (-57.7, 50.7) | 0.89 | -13.6 (-56.2, 29.1) | 0.53 | -25.3 (-68.5, 17.9) | 0.25 | -12.3 (60.5, 35.9) | 0.61 |
| % monocytes / PBMC | 0.7 (-1.2, 2.7) | 0.46 | 0.1 (-1.9, 2.0)  ♂ -0.6 (-5.1, 3.7)  ♀ 0.2 (-1.9, 2.2) | 0.96  ♂ 0.76  ♀ 0.89 | -0.4 (-1.8, 0.9) | 0.53 | -0.8 (-2.1, 0.6) | 0.25 | -0.6 (-2.1, 0.9) | 0.45 |
| B-cells^1^ | -24.1 (-111.1, 63.3) | 0.59 | -29.8 (119.2, 59.4) | 0.51 | -5.9 (-42.7, 30.9) | 0.75 | -12.3 (-47.8, 23.2) | 0.49 | -16.7 (57.9, 24.4) | 0.42 |
| % B-cells / lymfocytes | -1.00 (-3.39, 1.39) | 0.41 | -1.03 (-3.51, 1.45) | 0.41 | 0.10 (-0.41, 0.62) | 0.69 | 0.16 (-0.38, 0.69) | 0.56 | -0.01 (-0.57, 0.55) | 0.97 |
| NK-cells^1^ | -7.54 (-60.0, 44.9) | 0.78 | -36.7 (-114.2, 41.3) | 0.35 | 12.4 (-19.2, 43.9) | 0.44 | 5.5 (-26.6, 37.6) | 0.74 | 8.5 (-27.0, 43.9) | 0.64 |
| % NK-cells / lymfocytes | 1.0 (-1.0, 3.1) | 0.32 | 1.4 (-0.5, 3.2) | 0.14 | 0.6 (-0.3, 1.5) | 0.19 | 0.5 (-0.4, 1.4) | 0.29 | 0.5 (-0.6, 1.5) | 0.39 |
| T-cells^1^ | 21.3 (-327.4, 369.3) | 0.90 | -16.5 (-309.3, 276.2) | 0.91 | -39.7 (-191.2, 111.3) | 0.60 | -58.4 (-206.2, 89.5) | 0.44 | -62.6 (-226.3, 101.2) | 0.45 |
| % T-cells / lymfocytes | -0.72 (-3.96, 2.53) | 0.66 | -0.92 (-4.16, 2.33) | 0.57 | -0.42 (-1.39, 0.55) | 0.39 | -0.39 (-1.39, 0.59) | 0.43 | -0.32 (-1.42, 0.78) | 0.89 |
| CD4^+^ T-cells^1^ | 40.9 (-210.5, 292.9) | 0.75 | 13.2 (-201.3, 228.2) | 0.90 | -26.9 (-129,2, 75.8)  ♂ -134.5 (-283.3, 14.4)  ♀ 27.4 (-104.3, 159.2) | 0.60  ♂ 0.08  ♀ 0.68 | -41.6 (140.4, 56.9) | 0.40 | -44.9 (-155.5, 65.2) | 0.42 |
| % CD4^+^ T-cells | 0.73 (-3.97, 5.41) | 0.76 | 0.73 (-3.97, 5.42)  ♂ 4.26 (-5.60, 14.1)  ♀ -0.26 (-5.26, 4.85) | 0.76  ♂ 0.39  ♀ 0.92 | 0.05 (-0.79, 0.88)  ♂ 0.54 (-1.27, 2.34)  ♀ -0.18 (-1.14, 0.78) | 0.92  ♂ 0.55  ♀ 0.71 | 0.06 (-0.82, 0.94) | 0.89 | -0.07 (-1.04, 0.89) | 0.88 |
| CD8^+^ T-cells^1^ | -42.1 (-180.3, 95.8) | 0.55 | -50.4 (-162.3, 61.3) | 0.37 | -18.2 (-66.7, 30.4)  ♂ -0.3 (-1.85, 1.3)  ♀0.2 (-0.7, 1.0) | 0.46  ♂ 0.75  ♀ 0.73 | -24.8 (-72.8, 23.6) | 0.31 | -22.6 (-77.6, 32.4) | 0.42 |
| % CD8^+^ T-cells | -1.6 (-6.2, 3.1) | 0.50 | -1.8 (-6.2, 2.6)  ♂ -4.7 (-14.3, 4.8)  ♀ -0.9 (-5.7, 3.8) | 0.43  ♂ 0.32  ♀ 0.68 | -0.1 (-0.8, 0.7) | 0.98 | -0.1 (-0.8, 0.7) | 0.95 | 0.1 (-0.8, 0.9) | 0.86 |
| % DNT / T-cells | 0.7 (-0.5, 1.9) | 0.26 | 0.7 (-0.5, 1.9) | 0.27 | 0.1 (-0.2, 0.3) | 0.76 | 0.01 (-0.2, 0.3) | 0.71 | 0.1 (-0.2, 0.4) | 0.62 |
| %DPT / T-cells | 0.4 (-0.3, 1.0) | 0.28 | 1.3 (-1.7, 4.3) | 0.40 | 0.3 (-0.3, 0.9) | 0.35 | 0.3 (-0.4, 0.9) | 0.43 | 0.3 (-0.5, 1.0) | 0.45 |
| % CD56^+^ CD3^+^ T-cells | -1.16 (-3.05, 0.74) | 0.23 | -1.16 (-3.05, 0.74) | 0.23 | -0.05 (-0.37, 0.27) | 0.76 | -0.09 (-0.42, 0.25) | 0.59 | 0.04 (-0.31, 0.39) | 0.82 |
| % T-reg / CD4^+^ T-cells | -0.21 (-1.11, 0.68) | 0.64 | 0.11 (-0.73, 0.95) | 0.79 | 0.26 (-0.13, 0.64) | 0.19 | 0.32 (-0.06, 0.69) | 0.10 | 0.19 (-0.16, 0.56) | 0.28 |
| %CD25^+^ CD4^+^ T-cells | 2.30 (-4.43, 9.03) | 0.49 | 2.03 (-4.66, 8.73) | 0.55 | -0.15 (-1.58, 1.29) | 0.84 | -0.32 (-1.79, 1.15) | 0.67 | 0.05 (-1.55, 1.65) | 0.95 |
| %CD25^+^ CD8^+^ T-cells | 0.88 (-3.69, 5.46) | 0.70 | 1.27 (-3.58, 6.12) | 0.60 | 0.24 (-0.53, 1.00) | 0.53 | 0.23 (-0.57, 1.02) | 0.57 | 0.21 (-0.67, 1.09) | 0.64 |
| %HLA-DR^+^ T-cells | -1.37 (-3.86, 1.12) | 0.28 | -0.06 (-1.45, 1.33) | 0.93 | 0.37 (-0.04, 0.77) | 0.07 | 0.36 (-0.06, 0.78) | 0.09 | 0.37 (-0.08, 0.83) | 0.11 |
| %HLA-DR^+^ CD4^+^ T-cells / CD4^+^ T-cells | -0.35 (-1.74, 1.04) | 0.62 | -0.06 (-1.45, 1.33) | 0.93 | **0.28 (0.01, 0.55)** | **0.04** | 0.24 (-0.04, 0.52) | 0.09 | **0.37 (0.07, 0.66)** | **0.02** |
| %HLA-DR^+^ CD8^+^ T-cells / CD8^+^ T-cells | -2.95 (-7.24, 1.35) | 0.18 | -2.43 (-6.32, 1.47) | 0.22 | 0.20 (-0.39, 0.79) | 0.51 | 0.14 (-0.47, 0.76) | 0.65 | 0.06 (-0.59, 0.71) | 0.85 |
| % CD14^+^ / PBMC | 1.37 (-0.26, 2.99)  ♂ 1.14 (-2.47, 4.76)  ♀ 1.27 (-0.42, 2.96) | 0.10  ♂ 0.52  ♀ 0.14 | 0.98 (-0.96, 2.91)  ♂ 1.03 (-3.15, 4.21)  ♀ 0.74 (-1.32, 2.79) | 0.32  ♂ 0.62  ♀ 0.48 | -0.16 (-1.46, 1.13) | 0.80 | -0.36 (-1.68, 0.96) | 0.59 | -0.59 (-1.96, 0.78)  ♂ -0.71 (-2.91, 1.49)  ♀ -1.11 (-3.04, 0.83) | 0.39  ♂ 0.52  ♀ 0.26 |
| TLR4^+^ monocytes / CD14^+ 1^ | -0.01 (-0.04, 0.03) | 0.67 | -0.10 (-0.05, 0.03) | 0.59 | -0.01 (-0.02, 0.01) | 0.73 | -0.01 (-0.03, 0.01) | 0.52 | -0.01 (-0.03, 0.02) | 0.62 |
| HLA-DR^+^ monocytes / CD14^+ 1^ | 0.10 (-0.01, 0.02) | 0.11 | 0.01 (-0.003, 0.03) | 0.11 | 0.01 (-0.01, 0.01) | 0.69 | 0.01 (-0.01, 0.01) | 0.97 | -0.01 (-0.01, 0.01) | 0.73 |

^1^Absolute number: x10^9^/L. Sig. represents the p-value between the experimental intervention and control intervention.

Linear regression analysis was used to detect differences between the experimental and control intervention. Different models were tested: model 1: differences in baseline values; model 2: differences following the intervention values; model 3: differences following the intervention adjusted for baseline values; model 4: differences following the intervention adjusted for baseline, BMI, age and gender; and the final model 5: differences following the intervention values and adjusted for baseline, NDI score, PCS total score, kinesiophobia and the presence of central sensitisation.♂ effect modification for males; ♀ effect modification for females.

**Appendix 6** Differences in *ex-vivo* inflammatory markers between those with a good outcome versus poor outcome

|  | Time | B (95%CI:)  Model 1 | Sig. | Adjusted-B (95%CI:)  Model 2 | Sig. |
| --- | --- | --- | --- | --- | --- |
| hsCRP | T0 | -0.01 (-0.64, 0.62) | 0.97 | 0.01 (-0.62, 0.63) | 0.98 |
|  | T1 | -0.03 (-0.66, 0.60) | 0.92 | -0.01 (-0.64, 0.61) | 0.96 |
|  | T2 | -0.26 (-0.90, 0.37) | 0.42 | -0.26 (-0.89, 0.37) | 0.42 |
| TNF-α | T0 | -0.05 (-0.38, 0.28) | 0.76 | -0.07 (-0.42, 0.27) | 0.67 |
|  | T1 | 0.11 (-0.20, 0.41) | 0.49 | 0.08 (-0.23, 0.41) | 0.59 |
|  | T2 | 0.10 (-0.23, 0.42) | 0.55 | 0.07 (-0.26, 0.41) | 0.68 |
| TNF-RII | T0 | 0.15 (-0.16, 0.45) | 0.34 | 0.14 (-0.18, 0.46) | 0.39 |
|  | T1 | -0.01 (-0.45, 0.44) | 0.98 | -0.04 (-0.29, 0.42) | 0.87 |
|  | T2 | 0.18 (-0.13, 0.49) | 0.24 | 0.17 (-0.14, 0.49) | 0.29 |
| IL-1β | T0 | -0.14 (-0.77, 0.49) | 0.66 | -0.15 (-0.78, 0.47) | 0.63 |
|  | T1 | 0.10 (-0.51, 0.71) | 0.73 | 0.15 (-0.49, 0.79) | 0.64 |
|  | T2 | 0.07 (-0.32, 0.46) | 0.73 | 0.11 (-0.29, 0.51) | 0.59 |
| IL-1RA | T0 | 0.04 (-0.26, 0.33) | 0.81 | 0.04 (-0.26, 0.35) | 0.78 |
|  | T1 | 0.05 (-0.25, 0.34) | 0.74 | 0.06 (-0.20, 0.32) | 0.66 |
|  | T2 | 0.03 (-0.26, 0.33) | 0.82 | 0.04 (-0.23, 0.32) | 0.75 |
| Inflammatory index | T0 | 0.19 (-0.26, 0.75) | 0.49 | 0.18 (-0.37, 0.74) | 0.50 |
|  | T1 | 0.05 (-0.25, 0.34) | 0.75 | 0.02 (-0.27, 0.32) | 0.89 |
|  | T2 | 0.27 (-0.32, 0.87) | 0.37 | 0.27 (-0.32, 0.86) | 0.37 |
| Pro-inflammatory index | T0 | -0.05 (-0.32, 0.23) | 0.74 | -0.07 (-0.34, 0.19) | 0.58 |
|  | T1 | 0.09 (-0.19, 0.39) | 0.51 | 0.08 (-0.19, 0.37) | 0.54 |
|  | T2 | 0.02 (-0.23, 0.26) | 0.89 | 0.01 (-0.26, 0.27) | 0.99 |
| Anti-inflammatory index | T0 | 0.57 (-0.66, 1.80) | 0.35 | 0.55 (-0.71, 1.81) | 0.38 |
|  | T1 | -0.01 (-1.64, 1.63) | 0.99 | -0.11 (-1.76, 1.53) | 0.89 |
|  | T2 | 0.68 (-0.54, 1.91) | 0.27 | 0.64 (-0.60, 1.91) | 0.31 |
| Ratio pro/anti inflammatory index | T0 | 5.22 (-0.39, 10.8) | 0.07 | 5.22 (-0.67, 11.1) | 0.08 |
|  | T1 | -1.47 (-9.63, 6.68) | 0.72 | -1.52 (-9.71, 6.66) | 0.71 |
|  | T2 | 2.96 (-4.23, 10.2) | 0.41 | 3.26 (-4.09, 10.6) | 0.55 |

*Ex-vivo­* inflammatory markers were Ln-transformed. Sig. represents the p-value between the experimental intervention and control intervention. Mixed model analyses with fixed factor (time), covariate (group) and interaction (time*group) were used to detect differences between the groups at the three time moments. A random intercept was chosen to account for the correlated nature of multiple measurements from the same individual. T0: baseline; T1: immediate follow-up; T2: 2hours follow-up. Model 1: adjusted for plate number. Model 2: Additional adjusted for age; gender; body mass index. ♂ effect modification for males; ♀ effect modification for females.

**Appendix 7** Differences in *ex-vivo* cortisol between those with a good outcome versus poor outcome.

|  | B (95%CI:)  Model 1 | Sig. | B (95%CI:)  Model 2 | Sig. | Adjusted-B (95%CI:)  Model 3 | Sig. | Adjusted-B (95%CI:)  Model 4 | Sig. |
| --- | --- | --- | --- | --- | --- | --- | --- | --- |
| Serum Cortisol | -0.20 (-0.45, 0.05) | 0.11 | -0.17 (-0.40, 0.06) | 0.14 | -0.01 (-0.08, 0.06) | 0.74 | -0.002 (-0.06, 0.06) | 0.94 |

*Ex-vivo* cortisol was Ln-transformed. Sig. represents the p-value between those with a good outcome versus poor outcome. Linear regression analysis was used to detect differences between the experimental and control intervention. Different models were tested: model 1: differences in baseline values; model 2: differences following the intervention values; model 3: differences following the intervention adjusted for baseline values; model 4: differences following the intervention adjusted for baseline values, estrogen medication use and menstrual cycle (only applicable for serum cortisol).

**Appendix 8** Differences in inflammatory concentration after *in-vitro* stimulation of whole blood cells those with a good outcome versus poor outcome.

|  | Time | Stimulation | B (95%CI:)  Model 1 | Sig. | Norm. B  (95%CI:) Model 2 | Sig. | Norm. adjusted-B  (95%CI:) Model 3 | Sig. |
| --- | --- | --- | --- | --- | --- | --- | --- | --- |
| TNF-α | T0 | 1ng/ml | -0.01 (-0.47, 0.45) | 0.97 | 0.06 (-0.46, 0.57) | 0.82 | 0.02 (-0.52, 0.55) | 0.96 |
|  |  | 10µg/ml | 0.28, (-0.17, 0.74) | 0.22 | 0.34 (-0.18, 0.87) | 0.19 | 0.30 (-0.23, 0.84) | 0.26 |
|  | T1 | 1ng/ml | 0.19 (-0.27, 0.65) | 0.41 | 0.19 (-0.25, 0.64) | 0.19 | 0.17 (-0.30, 0.64) | 0.47 |
|  |  | 10µg/ml | 0.23 (-0.21, 0.67) | 0.29 | 0.31 (-0.16, 0.79) | 0.19 | 0.29 (-0.19, 0.79) | 0.23 |
|  | T2 | 1ng/ml | 0.27 (-0.27, 0.82) | 0.33 | 0.31 (-0.23, 0.86) | 0.26 | 0.29 (-0.28, 0.86) | 0.31 |
|  |  | 10µg/ml | 0.34 (-0.19, 0.86) | 0.21 | 0.41 (-0.12, 0.94) | 0.13 | 0.39 (-0.15, 0.94) | 0.24 |
| IL-1β | T0 | 1ng/ml | -0.33 (-0.86, 0.19) | 0.21 | -0.26 (-0.81, 0.28) | 0.34 | -0.19 (-0.74, 0.36) | 0.50 |
|  |  | 10µg/ml | -0.02 (-0.38, 0.34) | 0.91 | 0.04 (-0.39, 0.48) | 0.84 | 0.04 (-0.42, 0.49) | 0.87 |
|  | T1 | 1ng/ml | -0.11 (-0.61, 0.39) | 0.65 | -0.09 (-0.59, 0.39) | 0.69 | 0.02 (-0.48, 0.52) | 0.94 |
|  |  | 10µg/ml | 0.07 (-0.28, 0.43) | 0.69 | 0.14 (-0.25, 0.54) | 0.47 | 0.16 (-0.24, 0.56) | 0.42 |
|  | T2 | 1ng/ml | 0.14 (-0.46, 0.74) | 0.65 | 0.18 (-0.42, 0.77) | 0.56 | 0.28 (-0.32, 0.89) | 0.36 |
|  |  | 10µg/ml | -0.03 (-0.50, 0.44) | 0.91 | 0.05 (-0.41, 0.51) | 0.83 | 0.04 (-0.43, 0.52) | 0.86 |
| IL-1RA | T0 | 1ng/ml | **-0.24 (-0.47, -0.01)** | **0.04** | -0.17 (-0.47, 0.14) | 0.27 | -0.18 (-0.49, 0.13) | 0.24 |
|  |  | 10µg/ml | -0.01 (-0.21, 0.21) | 0.98 | 0.06 (-0.25, 0.38) | 0.70 | 0.07 (-0.23, 0.37) | 0.65 |
|  | T1 | 1ng/ml | -0.11 (-0.32, 0.08) | 0.26 | -0.06 (-0.31, 0.17) | 0.59 | -0.05 (-0.27, 0.17) | 0.63 |
|  |  | 10µg/ml | -0.01 (-0.21 (0.21) | 0.95 | 0.05 (-0.18, 0.29) | 0.77 | 0.07 (-0.14, 0.29) | 0.52 |
|  | T2 | 1ng/ml | 0.01 (0.32, 0.34) | 0.95 | 0.06 (-0.26, 0.39) | 0.69 | 0.08 (-0.22, 0.39) | 0.59 |
|  |  | 10µg/ml | -0.01 (0.24, 0.24) | 0.99 | 0.06 (-0.17, 0.30) | 0.59 | 0.08 (-0.14, 0.31) | 0.45 |
| IL-10 | T0 | 1ng/ml | -0.17 (-0.69, 0.34) | 0.51 | -0.11 (-0.62, 0.40) | 0.68 | -0.13 (-0.63, 0.37) | 0.60 |
|  |  | 10µg/ml | 0.10 (-0.32, 0.53) | 0.63 | 0.16 (-0.28, 0.61) | 0.47 | 0.11 (-0.31, 0.52) | 0.61 |
|  | T1 | 1ng/ml | -0.03 (-0.51, 0.44) | 0.89 | 0.01 (-0.42, 0.42) | 0.99 | -0.01 (-0.41, 0.38) | 0.94 |
|  |  | 10µg/ml | -0.03 (-0.51, 0.43) | 0.89 | 0.02 (-0.44, 0.49) | 0.92 | -0.03 (-0.47, 0.40) | 0.87 |
|  | T2 | 1ng/ml | 0.24 (-0.26, 0.75) | 0.34 | 0.28 (-0.20, 0.77) | 0.24 | 0.32 (-0.13, 0.78) | 0.16 |
|  |  | 10µg/ml | 0.23 (-0.21, 0.67) | 0.30 | 0.32 (-0.27, 0.91) | 0.29 | 0.32 (-0.24, 0.89) | 0.26 |
| CCL2 | T0 | 1ng/ml | 0.11 (-0.27, 0.47) | 0.59 | 0.17 (-0.25, 0.61) | 0.42 | 0.26 (-0.18, 0.69) | 0.25 |
|  |  | 10µg/ml | -0.32 (-0.70, 0.06) | 0.10 | -0.26 (-0.64, 0.11) | 0.17 | -0.21 (-0.59, 0.17) | 0.28 |
|  | T1 | 1ng/ml | -0.14 (-0.47, 0.18) | 0.37 | -0.06 (-0.41, 0.29) | 0.75 | 0.04 (-0.31, 0.39) | 0.80 |
|  |  | 10µg/ml | **-0.51 (-0.90, -0.13)** | **0.01** | **-0.49 (-0.83, -0.14)** | **0.007** | **-0.41 (-0.77, -0.07)** | **0.02** |
|  | T2 | 1ng/ml | -0.03 (-0.44, 0.39) | 0.90 | 0.05 (-0.39, 0.49) | 0.82 | 0.14 (-0.31, 0.59) | 0.52 |
|  |  | 10µg/ml | -0.35 (-0.77, 0.07) | 0.10 | -0.32 (-0.71, 0.07) | 0.11 | -0.25 (-0.65, 0.14) | 0.56 |
| CCL3 | T0 | 1ng/ml | -0.03 (-0.43, 0.37) | 0.89 | 0.04 (-0.41, 0.48) | 0.86 | 0.02 (-0.43, 0.48) | 0.91 |
|  |  | 10µg/ml | 0.13 (-0.19, 0.44) | 0.44 | 0.21 (-0.17, 0.59) | 0.27 | 0.18 (-0.21, 0.58) | 0.36 |
|  | T1 | 1ng/ml | 0.17 (-0.23, 0.56) | 0.41 | 0.18 (-0.21, 0.57) | 0.35 | 0.19 (-0.22, 0.59) | 0.36 |
|  |  | 10µg/ml | 0.04 (-0.27, 0.36) | 0.78 | 0.13 (-0.21, 0.47) | 0.56 | 0.11 (-0.25, 0.46) | 0.55 |
|  | T2 | 1ng/ml | 0.45 (-0.21, 1.11) | 0.18 | 0.49 (-0.16, 1.14) | 0.14 | 0.49 (-0.18, 1.16) | 0.15 |
|  |  | 10µg/ml | 0.18 (-0.13, 0.51) | 0.26 | 0.27 (-0.09 (0.64) | 0.14 | 0.24 (-0.13, 0.62) | 0.10 |
| CCL4 | T0 | 1ng/ml | 0.12 (-0.19, 0.44) | 0.43 | 0.14 (-0.20, 0.48) | 0.41 | 0.13 (-0.21, 0.49) | 0.44 |
|  |  | 10µg/ml | -0.05 (-0.35, 0.27) | 0.77 | 0.16 (-0.16, 0.47) | 0.33 | 0.15 (-0.18, 0.47) | 0.38 |
|  | T1 | 1ng/ml | 0.05 (-0.28, 0.37) | 0.78 | 0.06 (-0.23, 0.35) | 0.65 | 0.08 (-0.22, 0.38) | 0.62 |
|  |  | 10µg/ml | 0.04 (-0.26, 0.33) | 0.81 | 0.09 (-0.16, 0.33) | 0.47 | 0.08 (-0.17, 0.34) | 0.49 |
|  | T2 | 1ng/ml | 0.19 (-0.14, 0.51) | 0.26 | 0.32 (-0.17, 0.80) | 0.20 | 0.32 (-0.18, 0.82) | 0.42 |
|  |  | 10µg/ml | 0.27 (-0.26, 0.79) | 0.31 | 0.10 (-0.11, 0.33) | 0.35 | 0.09 (-0.13, 0.33) | 0.39 |
| Inflammatory index | T0 | 1ng/ml | -0.22 (-0.73, 0.30) | 0.41 | -0.02 (-0.47, 0.44) | 0.94 | -0.01 (-0.47, 0.45) | 0.97 |
|  |  | 10µg/ml | 0.03 (-0.28, 0.35) | 0.84 | 0.10 (-0.32, 0.52) | 0.63 | 0.08 (-0.34, 0.52) | 0.68 |
|  | T1 | 1ng/ml | -0.05 (-0.56, 0.46) | 0.47 | 0.04 (-0.31, 0.39) | 0.81 | 0.08 (-0.28, 0.45) | 0.66 |
|  |  | 10µg/ml | -0.06 (-0.41, 0.27) | 0.69 | -0.01 (-0.33, 0.32) | 0.96 | -0.01 (-0.33, 0.32) | 0.97 |
|  | T2 | 1ng/ml | 0.27 (-0.24, 0.79) | 0.29 | 0.32 (-0.20, 0.85) | 0.22 | 0.37 (-0.17, 0.91) | 0.18 |
|  |  | 10µg/ml | 0.06 (-0.32, 0.44) | 0.75 | 0.14 (-0.15, 0.44) | 0.33 | 0.15 (-0.14, 0.45) | 0.59 |
| Pro-inflammatory index | T0 | 1ng/ml | -0.07 (-0.47, 0.34) | 0.74 | 0.07 (-0.38, 0.51) | 0.75 | 0.09 (-0.36, 0.56) | 0.68 |
|  |  | 10µg/ml | 0.02 (-0.29, 0.34) | 0.89 | 0.08 (-0.33, 0.51) | 0.69 | 0.08 (-0.35, 0.50)  ♂ -0.41 (-1.07, 0.25)  ♀ 0.21 (-0.37, 0.79) | 0.74  ♂ 0.21  ♀ 0.47 |
|  | T1 | 1ng/ml | 0.04 (-0.36, 0.44) | 0.83 | 0.08 (-0.28, 0.46) | 0.65 | 0.14 (-0.24, 0.53) | 0.47 |
|  |  | 10µg/ml | -0.08 (-0.41, 0.25) | 0.62 | -0.03 (-0.35, 0.29) | 0.86 | -0.02 (-0.35, 0.31)  **♂ -0.77 (-1.42, -1.09)**  ♀ 0.21 (-0.18, 0.61) | 0.91  **♂ 0.02**  ♀ 0.28 |
|  | T2 | 1ng/ml | 0.33 (-0.31, 0.98) | 0.31 | 0.36 (-0.21, 0.94) | 0.21 | 0.42 (-0.17, 1.00) | 0.17 |
|  |  | 10µg/ml | 0.02 (-0.34, 0.39) | 0.91 | 0.08 (-0.21, 0.37) | 0.56 | 0.08 (-0.21, 0.38)  ♂ -0.47 (-1.02, 0.07)  ♀ 0.28 (-0.11, 0.66) | 0.57  ♂ 0.09  ♀ 0.41 |
| Anti-inflammatory index | T0 | 1ng/ml | -0.57, -1.18, 0.04) | 0.07 | -0.24 (-0.75, 0.27) | 0.36 | -0.25 (-0.77, 0.25) | 0.32 |
|  |  | 10µg/ml | 0.0-6 (-0.37, 0.51) | 0.77 | 0.17 (-0.32, 0.66) | 0.49 | 0.13 (-0.36, 0.62) | 0.59 |
|  | T1 | 1ng/ml | -0.24 (-0.82, 0.33) | 0.41 | -0.07 (-0.46, 0.32) | 0.73 | -0.06 (-0.43, 0.31) | 0.75 |
|  |  | 10µg/ml | -0.03 (-0.51, 0.44) | 0.89 | 0.06 (-0.36, 0.48) | 0.77 | 0.03 (-0.40, 0.45) | 0.90 |
|  | T2 | 1ng/ml | 0.16 (-0.64, 0.96) | 0.69 | 0.23 (-0.26, 0.72) | 0.36 | 0.27 (-0.21, 0.75) | 0.26 |
|  |  | 10µg/ml | 0.16 (-0.35, 0.67) | 0.53 | 0.29 (0.21, 0.79) | 0.24 | 0.31 (-0.40, 0.45) | 0.74 |
| Ratio pro/anti inflammatory index | T0 | 1ng/ml | -0.57 (-1.18, 0.04) | 0.07 | -3.01 (-7.07, 1.04) | 0.14 | -3.43 (-7.51, 0.65)  **♂ -12.32 (-24.82, 0.22)**  ♀ -0.27 (-1.32, 0.77) | 0.09  **♂ 0.05**  ♀ 0.60 |
|  |  | 10µg/ml | -0.11 (-1.97, 1.74) | 0.91 | -19.3 (-43.7, 5.19) | 0.12 | -19.0 (-43.8, 5.80) | 0.13 |
|  | T1 | 1ng/ml | -0.24 (-0.82, 0.33) | 0.41 | -3.16 (-7.57, 1.25) | 0.16 | -3.49 (-7.98, 0.99)  ♂ -1.38 (-10.1, 7.35)  ♀ -3.77 (-9.04, 1.50) | 0.12  ♂ 0.74  ♀ 0.16 |
|  |  | 10µg/ml | -0.84 (-1.77, 0.08) | 0.08 | -0.11 (-3.61, 1.49) | 0.41 | -0.81 (-3.45, 1.82) | 0.54 |
|  | T2 | 1ng/ml | 0.16 (-0.64, 0.96) | 0.70 | -0.76 (-3.62, 2.08) | 0.59 | -1.21 (-4.12, 1.69)  ♂ -5.05 (-13.0, 2.93)  ♀ 0.54 (-1.88, 2.98) | 0.40  ♂ 0.20  ♀ 0.65 |
|  |  | 10µg/ml | -10.5 (-34.6, 13.6) | 0.39 | -0.11 (-2.43, 2.29) | 0.92 | 0.11 (-2.25, 2.46) | 0.93 |

*In-vitro­* inflammatory markers were Ln-transformed. Sig. represents the p-value between those with a good outcome versus poor outcome. Mixed model analyses with fixed factor (time), covariate (group) and interaction (time*group) were used to detect differences between the groups at the three time moments. A random intercept was chosen to account for the correlated nature of multiple measurements from the same individual. T0: baseline; T1: immediate follow-up; T2: 2hours follow-up. Model 1: adjusted for plate number. Model 2: additional normalised /1000 monocytes. Model 3: Additional adjusted for age; gender; body mass index. ♂ effect modification for males; ♀ effect modification for females.

**Appendix 9** Differences in cell staining and blood phenotyping between those with a good outcome versus poor outcome

| PBMC Phenotype | B (95%CI:) model 1 | Sig. | B (95%CI:) model 2 | Sig. | B (95%CI:) model 3 | Sig. | B (95%CI:) model 4 | Sig. |
| --- | --- | --- | --- | --- | --- | --- | --- | --- |
| Leucocytes^1^ | -0.81 (-2.01, 0.40) | 0.19 | -0.57 (-1.75, 0.61) | 0.33 | 0.06 (-0.50, 0.61) | 0.84 | 0.09 (-0.52, 0.71) | 0.75 |
| PBMC^1^ | -343 (-968, 283) | 0.28 | -283 (-772, 206) | 0.25 | -74 (-345, 197) | 0.59 | -61 (-344, 221) | 0.66 |
| Lymfocytes^1^ | -287 (-867, 291) | 0.33 | -243 (-698, 212) | 0.29 | -65 (-305, 175) | 0.59 | -63 (-307, 182) | 0.61 |
| Monocytes (scatter)^1^ | -8.9 (-84.5, 66.6) | 0.81 | -8.3 (-73.6, 57.0) | 0.80 | -2.1 (-53.7, 49.5) | 0.94 | 1.0 (-54.2, 56.3) | 0.97 |
| % monocytes / PBMC | 0.79 (-1.83, 3.79) | 0.49 | 0.93 (-1.42, 3.28) | 0.43 | 0.41 (-1.15, 1.96) | 0.60 | 0.37 (-1.14, 1.87) | 0.63 |
| B-cells^1^ | -85.3 (-201, 31.1) | 0.15 | -96.4 (-220.4, 27.3) | 0.12 | -12.8 (-71.8, 46.2) | 0.66 | -9.96 (-69.5, 49.6) | 0.74 |
| % B-cells / lymfocytes | -1.62 (-4.39, 1.16) | 0.25 | -1.89 (-5.01, 1.22) | 0.23 | 0.20 (-0.54, 0.94) | 0.59 | 0.16 (-0.66, 0.97) | 0.70 |
| NK-cells^1^ | 7.54 (-42.7, 57.8) | 0.77 | 23.7 (-12.7, 56.1) | 0.49 | -4.93 (-37.6, 27.8) | 0.76 | -1.63 (-34.9, 31.8) | 0.92 |
| % NK-cells / lymfocytes | 2.34 (-0.12, 4.79) | 0.06 | 1.94 (-0.21, 4.09) | 0.08 | 0.33 (-0.91, 1.56) | 0.60 | 0.55 (-0.76, 1.86) | 0.40 |
| T-cells^1^ | -237 (-733, 257) | 0.34 | -168 (-546, 209) | 0.38 | -27.4 (-209, 155) | 0.76 | -23.2 (-209, 163) | 0.80 |
| % T-cells / lymfocytes | -1.86 (-6.09, 2.36) | 0.38 | -1.21 (-5.36, 2.93) | 0.56 | 0.14 (-1.16, 1.43) | 0.84 | 0.10 (-1.32, 1.52) | 0.89 |
| CD4^+^ T-cells^1^ | -255 (-610, 100) | 0.16 | -179 (-464, 106) | 0.21 | -16.1 (-148, 115) | 0.81 | -19.7 (-150, 111) | 0.76 |
| % CD4^+^ T-cells | -3.86 (-9.63, 1.91) | 0.19 | -3.86 (-9.63, 1.91) | 0.19 | 0.03 (-1.12, 1.67)  ♂ -0.23 (-2.75, 2.29)  ♀ 0.11 (-1.25, 1.47) | 0.96  ♂ 0.85  ♀ 0.87 | -0.21 (-1.47, 1.05) | 0.74 |
| CD8^+^ T-cells^1^ | 21 (-167, 209) | 0.83 | 4 (-135, 144) | 0.95 | -8 (-63, 47)  ♂ 37 (-94, 168)  ♀ -25 (-90, 39.1) | 0.75  ♂ 0.56  ♀ 0.42 | -6 (-63, 52) | 0.84 |
| % CD8^+^ T-cells | 3.82 (-2.26, 9.91) | 0.21 | 3.15 (-2.55, 8.86) | 0.27 | -0.21 (-1.25, 0.84)  ♂ -0.45 (-2.75, 1.85)  ♀ -0.10 (-1.33, 1.14) | 0.69  ♂ 0.69  ♀ 0.87 | 0.09 (-1.03, 1.23) | 0.87 |
| % DNT / T-cells | -0.21 (-1.28, 0.86) | 0.69 | -0.16 (-1.22, 0.89) | 0.76 | 0.13 (-0.16, 0.43) | 0.37 | 0.07 (-0.25, 0.38) | 0.67 |
| % DPT / T-cells | 0.87 (-0.22, 1.96) | 0.12 | -2.94 (-8.25, 2.38) | 0.27 | 1.00 (-0.08, 2.09) | 0.07 | **1.24 (0.06, 2.43)** | **0.04** |
| % CD56^+^ CD3^+^ T-cells | 0.80 (-1.48, 30.8) | 0.49 | 0.63 (-1.68, 2.94) | 0.59 | -0.11 (-0.49, 0.27) | 0.56 | -0.13 (-0.55, 0.29) | 0.53 |
| % T-reg / CD4^+^ T-cells | **1.27 (0.23, 2.30)**  ♂ -0.13 (-2.57, 2.31)  **♀ 1.61 (0.36, 2.86)** | **0.02**  ♂ 0.91  **♀ 0.01** | 0.88 (-0.26, 2.03) | 0.13 | 0.46 (-0.03, 0.94) | 0.07 | 0.35 (-0.17, 0.88) | 0.19 |
| %CD25^+^ CD4^+^ T-cells | 1.25 (-7.21, 9.72) | 0.76 | -1.30 (-9.81, 7.20) | 0.76 | **-2.44 (-4.64, -0.25)** | **0.03** | **-2.29 (-4.64, 0.05)** | **0.06** |
| %CD25^+^ CD8^+^ T-cells | 0.86 (-4.59, 5.30) | 0.69 | 0.36 (-3.51, 5.23) | 0.88 | -0.77 (-1.86, 0.32) | 0.16 | -0.98 (-2.15, 0.18) | 0.09 |
| %HLA-DR^+^ T-cells | **3.26 (0.88, 5.65)** | **0.008** | 0.16 (-0.29, 0.61) | 0.49 | 0.16 (-0.29, 0.61) | 0.49 | 0.19 (-0.29, 0.67) | 0.43 |
| %HLA-DR^+^ CD4^+^ T-cells / CD4^+^ T-cells | **1.94 (0.77, 3.12)**  ♂ 2.22 (-0.57, 5.01)  **♀ 1.65 (0.38, 2.93)** | **0.002**  ♂ 0.11  **♀ 0.01** | **1.91 (0.77, 3.04)** | **0.001** | 0.11 (-0.26, 0.49) | 0.55 | 0.11 (-0.28, 0.51) | 0.57 |
| %HLA-DR^+^ CD8^+^ T-cells / CD8^+^ T-cells | **5.45 (0.24, 10.6)** | **0.04** | **5.24 (0.54, 9.95)**  ♂ 7.17 (-6.73, 21.0)  **♀ 3.18 (0.31, 6.06)** | **0.03**  ♂ 0.29  **♀ 0.03** | 0.37 (-0.36, 1.09) | 0.31 | 0.38 (-0.39, 1.14) | 0.33 |
| % CD14^+^ / PBMC | 2.01 (-0.63, 4.65) | 0.13 | 1.66 (-0.63, 4.65)  ♂ 1.22 (-5.04, 7.48)  ♀ 1.54 (-1.15, 4.23) | 0.16  ♂ 0.69  ♀ 0.25 | 0.74 (-1.00, 2.49)  ♂ 0.88 (-2.73, 4.49)  ♀ 0.41 (-1.59, 2.43) | 0.39  ♂ 0.61  ♀ 0.68 | 0.75 (-1.02, 2.51) | 0.40 |
| TLR4^+^ monocytes / CD14^+ 1^ | 0.02 (-0.68, 3.99) | 0.36 | 0.01 (-0.02, 0.06) | 0.65 | -0.009 (-0.04, 0.02) | 0.59 | -0.01 (-0.04, 0.02) | 0.42 |
| HLA-DR^+^ monocytes / CD14^+ 1^ | -0.009 (-0.04, 0.07) | 0.22 | -0.01 (-0.03, 0.006) | 0.17 | 0.001 (-0.007, 0.006) | 0.90 | -0.001 (-0.008, 0.006) | 0.85 |

^1^Absolute number: x10^9^/L. Sig. represents the p-value between the those with a good outcome versus poor outcome

Linear regression analysis was used to detect differences between the experimental and control intervention. Different models were tested: model 1: differences in baseline values; model 2: differences following the intervention values; model 3: differences following the intervention adjusted for baseline values; model 4: differences following the intervention adjusted for baseline, BMI, age and gender. ♂ effect modification for males; ♀ effect modification for females.

**Appendix 10** Association between the change in neuroimmune response and pain intensity in the experimental group

|  | Time | Stimulation | Regression coefficient B (95%CI:) | Sig. |
| --- | --- | --- | --- | --- |
| *Ex-vivo* Cortisol | T1 |  | 0.001 (0.000, 0.003) | 0.16 |
| *Ex-vivo* IL-1β | T1 |  | 0.006 (-0.01, 0.02) | 0.47 |
|  | T2 |  | -0.005 (-0.01, 0.004) | 0.24 |
| *Ex-vivo* TNF-α | T1 |  | -0.004 (-0.007, 0.000) | 0.05 |
|  | T2 |  | -0.002 (-0.006, 0.003) | 0.43 |
| *Ex-vivo* IL-1RA | T1 |  | -0.001 (-0.004, 0.003) | 0.70 |
|  | T2 |  | -0.001 (-0.005, 0.003) | 0.51 |
| *Ex-vivo* hsCRP | T1 |  | 0.001 (-0.002, 0.002) | 0.69 |
|  | T2 |  | 0.003 (-0.001, 0.006) | 0.14 |
| *Ex-vivo* TNFR2 | T1 |  | 0.001 (-0.003, 0.004) | 0.87 |
|  | T2 |  | -0.001 (-0.002, 0.001) | 0.56 |
| *In-vitro IL-1RA* | T1 | 1ng/ml | 0.002 (-0.003, 0.009) | 0.31 |
|  |  | 10µ/ml | 0.003 (-0.002, 0.009) | 0.27 |
|  | T2 | 1ng/ml | -0.001 (-0.009, 0.007) | 0.81 |
|  |  | 10µ/ml | 0.001 (-0.006, 0.006) | 0.89 |
| *In-vitro IL-1β* | T1 | 1ng/ml | 0.003 (-0.008, 0.013) | 0.62 |
|  |  | 10µ/ml | 0.004 (-0.004, 0.012) | 0.35 |
|  | T2 | 1ng/ml | -0.007 (-0.019, 0.005) | 0.23 |
|  |  | 10µ/ml | -0.001 (-0.010, 0.007) | 0.75 |
| *In-vitro TNF-α* | T1 | 1ng/ml | 0.001 (-0.006, 0.008) | 0.83 |
|  |  | 10µ/ml | 0.003 (-0.004, 0.010) | 0.38 |
|  | T2 | 1ng/ml | -0.002 (-0.013, 0.008) | 0.62 |
|  |  | 10µ/ml | -0.002 (-0.011, 0.008) | 0.74 |
| *In-vitro IL-10* | T1 | 1ng/ml | 0.001 (-0.008, 0.010) | 0.83 |
|  |  | 10µ/ml | 0.005 (-0.003, 0.014) | 0.23 |
|  | T2 | 1ng/ml | 0.007 (-0.017, 0.003 | 0.16 |
|  |  | 10µ/ml | -0.006 (-0.016, 0.005) | 0.29 |
| *In-vitro CCL2* | T1 | 1ng/ml | 0.004 (-0.003, 0.011) | 0.29 |
|  |  | 10µ/ml | **0.008 (0.001, 0.014)** | **0.02** |
|  | T2 | 1ng/ml | 0.005 (-0.004, 0.013) | 0.31 |
|  |  | 10µ/ml | 0.005 (-0.003, 0.013) | 0.19 |
| *In-vitro CCL3* | T1 | 1ng/ml | 0.001 ((-0.007, 0.007)  ♂ 0.002 (-0.012, 0.016)  ♀ -0.006 (-0.014, 0.003) | 0.96  ♂ 0.73  ♀ 0.17 |
|  |  | 10µ/ml | 0.005 (-0.002, 0.011) | 0.13 |
|  | T2 | 1ng/ml | -0.004 (-0.018, 0.009) | 0.51 |
|  |  | 10µ/ml | -0.003 (-0.010, 0.005) | 0.47 |
| *In-vitro CCL4* | T1 | 1ng/ml | 0.003 (-0.003, 0.010)  ♂ 0.004 (-0.007, 0.015)  ♀ -0.001 (-0.010, 0.007) | 0.32  ♂ 0.49  ♀ 0.79 |
|  |  | 10µ/ml | 0.004 (-0.002, 0.009) | 0.17 |
|  | T2 | 1ng/ml | -0.002 (-0.013, 0.009) | 0.68 |
|  |  | 10µ/ml | -0.001 (-0.006, 0.004) | 0.63 |

Sig. represents the p-value for the association between the pain scores and inflammatory markers. Linear regression analysis was used to assess the association between pain scores and inflammatory markers. Following the intervention inflammatory markers and following the intervention VAS scores were associated with the baseline inflammatory markers; baseline VAS scores and plate number as covariate. ♂ effect modification for males; ♀ effect modification for females. T1: difference between T0 baseline compared to immediate follow-up; T2: difference between T0 baseline compared to 2hours follow-up Bold represents a significant difference between groups (p<0.05)

**Appendix 11** Association between the change in neuroimmune response and change in pain intensity in the experimental group

|  | Time |  | Regression coefficient B (95%CI:) | Sig. |
| --- | --- | --- | --- | --- |
| *Ex-vivo* inflammatory index | T1 |  | 0.001 (-0.004, 0.007)  ♂ 0.004 (-0.004, 0.014)  ♀ 0.001 (-0.008, 0.007) | 0.64  ♂ 0.27  ♀ 0.89 |
|  | T2 |  | -0.003 (-0.008, 0.002) | 0.24 |
| *Ex-vivo* pro-inflammatory index | T1 |  | -0.001 (-0.007, 0.005) | 0.69 |
|  | T2 |  | -0.003 (-0.008, 0.003) | 0.32 |
| *Ex-vivo* anti-inflammatory index | T1 |  | 0.002 (-0.008, 0.011) | 0.74 |
|  | T2 |  | -0.003 (-0.010, 0.004) | 0.36 |
| *Ex-vivo* ratio pro/anti-inflammatory index | T1 |  | -0.05 (-0.127, 0.025) | 0.19 |
|  | T2 |  | -0.003 (-0.080, 0.074) | 0.94 |
| *In-vitro* inflammatory index | T1 | 1ng/ml | 0.004 (-0.005, 0.012)  ♂ 0.003 (-0.016, 0.023)  ♀ -0.002 (-0.012, 0.008) | 0.42  ♂ 0.72  ♀ 0.72 |
|  |  | 10µ/ml | 0.008 (-0.003, 0.018) | 0.14 |
|  | T2 | 1ng/ml | -0.004 (-0.16, 0.008) | 0.47 |
|  |  | 10µ/ml | -0.004 (-0.014, 0.006) | 0.40 |
| *In-vitro* pro-inflammatory index | T1 | 1ng/ml | 0.004 (-0.005, 0.013) | 0.37 |
|  |  | 10µ/ml | 0.008 (-0.002, 0.018) | 0.12 |
|  | T2 | 1ng/ml | -0.003 (-0.017, 0.010) | 0.62 |
|  |  | 10µ/ml | -0.003 (-0.013, 0.008) | 0.61 |
| *In-vitro* anti-inflammatory index | T1 | 1ng/ml | 0.003 (-0.007, 0.012) | 0.59 |
|  |  | 10µ/ml | 0.008 (-0.005, 0.020) | 0.23 |
|  | T2 | 1ng/ml | -0.006 (-0.017, 0.004) | 0.24 |
|  |  | 10µ/ml | -0.007 (-0.021, 0.007) | 0.32 |
| *In-vitro* ratio pro/anti-inflammatory index | T1 | 1ng/ml | 0.012 (-0.026, 0.055) | 0.46 |
|  |  | 10µ/ml | 0.001 (-0.083, 0.083) | 0.99 |
|  | T2 | 1ng/ml | 0.570 (-0.043, 1.187) | 0.07 |
|  |  | 10µ/ml | -0.030 (-0.109, 0.601) | 0.56 |

Sig. represents the p-value for the association between the pain scores and inflammatory indices. Linear regression analysis was used to assess the association between pain scores and inflammatory indices. Following the intervention indices and following the intervention VAS scores were associated with the baseline inflammatory indices; baseline VAS scores and plate number as covariate. ♂ effect modification for males; ♀ effect modification for females. T1: difference between T0 baseline compared to immediate follow-up; T2: difference between T0 baseline compared to 2hours follow-up

**Appendix 12** Global Perceived Effect scores


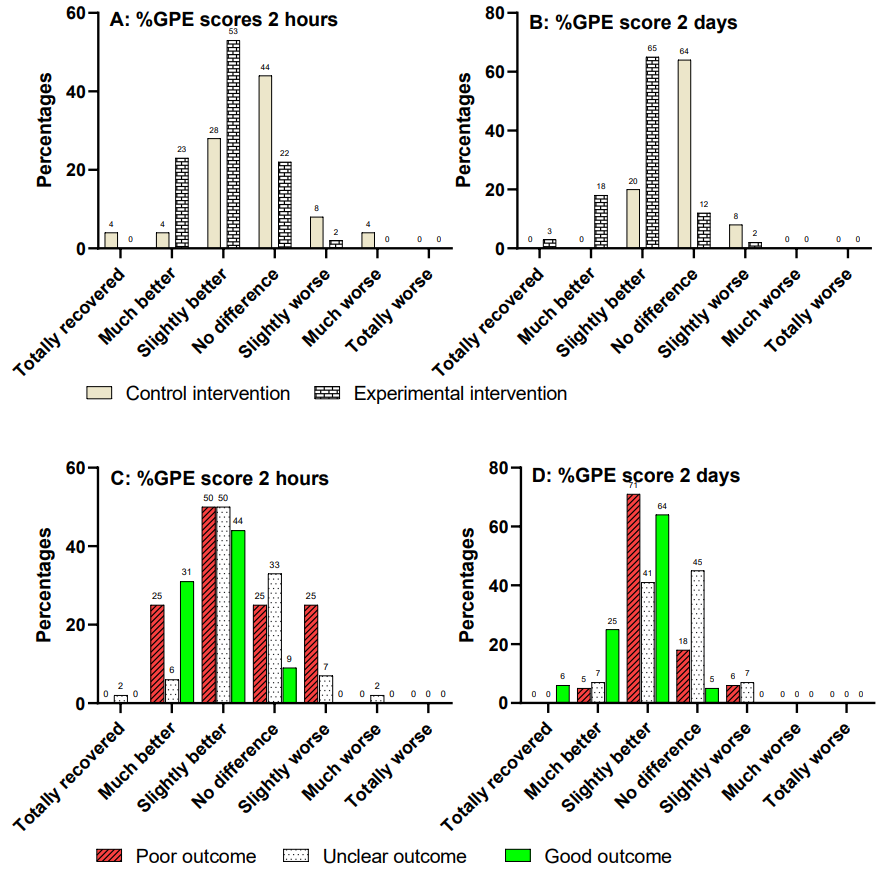


Visual illustration of the Global Perceived Effect (GPE-7) scores over time. Panel A-B. Percentages GPE scores between the control intervention and the experimental intervention at follow-up time points 2 hours and 2 days following the intervention. Panel C-D. Percentages GPE scores between poor -, unclear-, and good outcome at follow-up time points 2 hours and 2 days following the intervention.
